# Prioritising primary care respiratory research needs: results from the 2020 International Primary Care Respiratory Group (IPCRG) global e-Delphi exercise

**DOI:** 10.1038/s41533-021-00266-4

**Published:** 2022-01-28

**Authors:** Arwa Abdel-Aal, Karin Lisspers, Siân Williams, Peymané Adab, Rachel Adams, Dhiraj Agarwal, Amanda Barnard, Izolde Bouloukaki, Job F. M. van Boven, Niels Chavannes, Andrew P. Dickens, Frederik van Gemert, Mercedes Escarrer, Shamil Haroon, Alex Kayongo, Bruce Kirenga, Janwillem W. H. Kocks, Daniel Kotz, Chris Newby, Cliodna McNulty, Esther Metting, Luis Moral, Sophia Papadakis, Hilary Pinnock, David Price, Dermot Ryan, Sally J. Singh, Jaime Correia de Sousa, Björn Ställberg, Stanley J. Szefler, Stephanie J. C. Taylor, Ioanna Tsiligianni, Alice Turner, David Weller, Osman Yusuf, Aizhamal K. Tabyshova, Rachel E. Jordan

**Affiliations:** 1 Academy of Primary Care, University of Hull York Medical School, York, UK; 2 Department of Public Health and Caring Sciences, Family Medicine and Preventive Medicine, Uppsala University, Uppsala, Sweden; 3 Joint Chief Executive Officer, International Primary Care Respiratory Group, London, UK; 4 Institute of Applied Health Research, University of Birmingham, Birmingham, UK; 5 Vadu Rural Health Program, KEM Hospital Research Centre, Pune, India; 6 Charles Strut University and Australian National University, Canberra, Australia; 7 University of Crete, Medical School, Heraklion, Greece; 8 University of Groningen, University Medical Centre Groningen, Groningen, The Netherlands; 9 Leiden University Medical Center, Department of Public Health and Primary Care, Leiden, The Netherlands; 10 Spanish Pediatric Society of Clinical Immunology, Asthma and Allergy, Valencia, Spain; 11 Pediatric Allergy Service, Clinica Juaneda, Majorca, Spain; 12 Makerere University Lung Institute, Kampala, Uganda; 13 General Practitioners Research Institute, Groningen, The Netherlands; 14 Institute of General Practice, Medical Faculty of the Heinrich-Heine-University Düsseldorf, Düsseldorf, Germany; 15 Medical School, University of Nottingham, Nottingham, UK; 16 Primary Care and Interventions Unit, Gloucester, Public Health England, Gloucester, UK; 17 Alicante University General Hospital, Alicante Institute for Health and Biomedical Research (ISABIAL), Alicante, Spain; 18 Department of Social Medicine, University of Crete, Heraklion, Greece; 19 Allergy and Respiratory Group, Usher institute, University of Edinburgh, Edinburgh, UK; 20 Observational and Pragmatic Research Institute, Singapore, Singapore; 21 Centre of Academic Primary Care, Division of Applied Health Sciences, University of Aberdeen, Aberdeen, UK; 22 Pulmonary and Cardiac Rehabilitation, University of Leicester, Leicester, UK; 23 University of Minho, Braga, Portugal; 24 Paediatrics-Pulmonary Medicine, University of Colorado, Boulder, USA; 25 Institute of Population Health Sciences, Barts and the London School of Medicine, Queen Mary, University of London, London, UK; 26 The Allergy and Asthma Institute, Islamabad, Pakistan; 27 National Center of Cardiology and Internal Medicine, Bishkek, Kyrgyzstan

**Keywords:** Asthma, Chronic obstructive pulmonary disease, Health policy

## Abstract

Respiratory diseases remain a significant cause of global morbidity and mortality and primary care plays a central role in their prevention, diagnosis and management. An e-Delphi process was employed to identify and prioritise the current respiratory research needs of primary care health professionals worldwide. One hundred and twelve community-based physicians, nurses and other healthcare professionals from 27 high-, middle- and low-income countries suggested 608 initial research questions, reduced after evidence review by 27 academic experts to 176 questions covering diagnosis, management, monitoring, self-management and prognosis of asthma, COPD and other respiratory conditions (including infections, lung cancer, tobacco control, sleep apnoea). Forty-nine questions reached 80% consensus for importance. Cross-cutting themes identified were: a need for more effective training of primary care clinicians; evidence and guidelines specifically relevant to primary care, adaption for local and low-resource settings; empowerment of patients to improve self-management; and the role of the multidisciplinary healthcare team.

## Introduction

Chronic respiratory diseases (CRDs) impose a significant burden on global health^1^. The Global Burden of Disease (GBD) Study 2019 suggested that respiratory conditions account for 7.7 million deaths per year^1^; CRD and respiratory infections (including tuberculosis) account for the third and fourth causes of death after cardiovascular disease and cancer^2,3^. Furthermore, the number of Disability Adjusted Life Years (DALYs) for CRD has increased by 20% since 1990^2,3^. Tobacco smoking, the leading cause of CRD, is the second most important risk factor for global disease burden while indoor and outdoor air pollution are included in the top ten risk factors^2^. Commentaries by the GBD highlight the gap between current policies, activity and burden and the importance of universal health coverage^3^.

Primary care has a core role in the prevention, diagnosis and management of all respiratory diseases^4^; indeed, respiratory symptoms are the most common reason for primary care consultations^5^. However, significant evidence gaps remain, with a corresponding lack of evidence-based guidelines, quality standards and training to support primary care practice^5,6^. Progress is further challenged by the diversity of healthcare issues presented in primary care and the various models adopted for primary care worldwide^5^. Prioritising research needs helps guide researchers, research funders, and policymakers and will ultimately improve clinical guidelines and patient care globally^7^. Although relevant prioritisation studies exist^8,9^, there is still a need for a systematic and transparent approach in the specific area of primary care respiratory research^7^, and furthermore to ensure that the priorities are relevant to countries with different risk factor profiles and phases of development^10^. To date, there has been a general lack of investment in primary care respiratory research and an up-to-date specific needs statement will provide impetus to redress that balance^11^.

The International Primary Care Respiratory Group (IPCRG) is a clinically-led charity that aims to promote research into the care, management and prevention of respiratory diseases in the community^12^. Its vision is a “world breathing and feeling well through universal access to right care”. Current membership includes 37 full and 24 associate member countries^12^ representing an estimated 155,000 primary healthcare professionals worldwide from high-, middle- and low-income countries in Europe, Asia, North and South America, Australia, and Africa^13^. In 2010, the IPCRG published its first Research Needs Statement for primary care respiratory research, identifying 145 research questions within five domains: asthma, rhinitis, COPD, smoking and respiratory infections^6^. This was prioritised in 2012 through an e-Delphi exercise culminating in a final list of 62 questions^14^. Now, 8 years on, changing needs and contexts require an update.

In this paper, we provide a new agenda for primary care respiratory research, obtaining consensus on the most important respiratory research questions from the perspective of practising primary care healthcare professionals representing a wide range of backgrounds and settings worldwide.

## Methods

### Overview of the e-Delphi processes

**Fig. 1:**
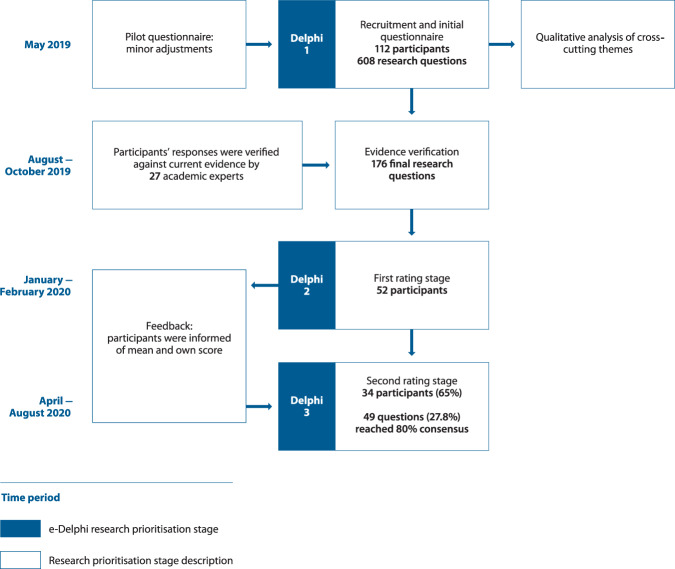
Flow diagram of the research prioritisation process.

An e-Delphi exercise with three rounds was undertaken to build consensus on the most important priorities for respiratory research in primary care^15,16^ (Fig. 1). It commenced in May 2019 and was completed in August 2020 and included research questions suggested by practising primary healthcare professionals from across the world, with input from a panel of experts to verify and refine these questions, and two further rounds to rate the priorities. In addition, the open comments from the first Delphi round were analysed qualitatively to identify cross-cutting themes.

### Recruitment

National coordinators from all IPCRG member countries were asked to purposively select and invite (by email) clinicians (doctors, nurses and any other healthcare professionals) working with respiratory patients in community settings in their countries to represent a broad range of views and experience. Specific inclusion criteria included the ability to complete online surveys in English and working in/with primary care settings to deliver care to patients with respiratory conditions.

### e-Delphi 1: initial open-ended questionnaire

All data were collected through the Jisc Online Survey tool^17^ The initial questionnaire included three open questions seeking opinions on the most common respiratory conditions encountered in their clinical practice; the most clinically important conditions (in terms of burden and impact) and to suggest research questions relevant to their stated conditions for which they perceived evidence to be lacking. Participants were asked to consider the following domains: diagnosis, management, monitoring, self-management and prognosis. This questionnaire was piloted for clarity and ease of use by members of the IPCRG Research Committee and amended accordingly.

Round 1 open-ended questionnaire to participants 1. What are the most common respiratory conditions encountered in your clinical practice? Please list 5–8 conditions.

2. Among those, which conditions are the most clinically important in your daily clinical practice (please consider the burden and impact of these conditions)?

3. Please list 10 questions relevant to the above conditions that you would like to see answered but currently cannot find enough evidence for them in the literature? Please carefully consider the following areas: diagnosis, management, monitoring, self-management and prognosis.

### Evidence verification stage

To ensure that the questions suggested by participants reflected genuine evidence gaps and were answerable as research questions, 27 academic experts (Supplementary Table 1) with topic-specific expertise related to primary care, associated with the IPCRG, reviewed and verified evidence against the questions suggested by participants, refining and grouping similar questions, removing duplicates and adding questions where appropriate (including referring to unanswered questions from the previous prioritisation exercise^5,14^) to produce a final list of relevant and answerable questions. Experts were provided with instructions and a standardised proforma to produce the modified final questions. Additionally, they were asked to provide justification and evidence from the literature in support of their final list of questions.

### e-Delphi round 2: first rating stage

All participants from the e-Delphi round 1 were invited to rate each question from the final list of research questions through two e-Delphi rating stages. During the first rating stage, participants rated each question on a 5-point Likert scale from 1 to 5 based on clinical importance (1 = Not at all important to 5 = Very important).

### e-Delphi round 3: second rating stage

All participants from the e-Delphi round 2 were invited to re-rate the same list of questions from the previous round. At this stage, each participant was asked to consider the mean score, their individual score and any justification/comments provided by the participants on the questions in e-Delphi round 2, before re-rating the questions. Consensus for the e-Delphi was defined in round 3 for any question when 80% or more of participants rated it as 4 or 5 (important or very important).

### Statistical analysis

Descriptive statistics were used to present the characteristics of participants and responses. Treemap charts were used to present the relative proportions of conditions mentioned in the questions. All questions were ranked by consensus score within three main topics: Asthma, COPD and Other, and within each topic, further ranked within 5 domains: prevention, diagnosis, management, self-management, monitoring and prognosis. The mean rating score was used in the final ranking to separate questions with the same consensus score. In the few cases where the consensus score and the mean rank score were identical, questions were listed in alphabetical order. All analyses were carried out using the analysis functions in the Jisc Online Survey *tool* and Microsoft Excel.

### In-depth qualitative analysis of cross-cutting themes from the initial questionnaire

The qualitative analysis focussed on the raw open-ended research questions received in the initial questionnaire and aimed to highlight cross-cutting needs, issues and possible solutions relevant to the care of respiratory patients in primary care. Thematic analysis was carried out by AAA using NVIVO 12 software. Three other authors (RJ, PA, KL) independently reviewed the data, which was followed by a discussion between these four authors to reach an agreement on the final themes.

### Ethics

This study was approved by the University of Birmingham Ethics Committee (ERN_19-0303B). The study complied with all relevant ethical regulations for work with human participants, and informed consent was obtained from all participants at the start of the online survey.

### Reporting summary

Further information on research design is available in the Nature Research Reporting Summary linked to this article.

## Results

### Participants

A total of 112 participants from 27 countries took part in the initial online e-Delphi survey. Participants came from a wide range of backgrounds, roles, and experiences (see Table 1). Participants represented all main global regions including Europe (*n* = 46, 41%), Asia (*n* = 37, 33%), Africa (*n* = 14, 12.5%), South America (*n* = 9, 8%), North America (*n* = 3, 2.7%) and Oceania (*n* = 3, 2.7%). There were similar numbers of high-income and low- and middle-income countries (LMICs) represented, but with a higher proportion of participants from LMICs (*n* = 67, 60%). Supplementary Table 2 provides further detail on the distribution of participants within the high-, middle- and low-income countries.

**Table 1:** Demographic characteristics of participants for e-Delphi rounds 1, 2 and 3.

e-Delphi round	Round 1	Round 2	Round 3
Characteristic	*N* (%)	*N* (%)	*N* (%)
Number of participants	112 (100.0)	52 (100.0)	34 (100.0)
Gender			
Male	47 (42.0)	21 (40.4)	12 (35.0)
Female	65 (58.0)	31 (59.6)	22 (65.0)
Age in years			
25–34	28 (25.0)	14 (27.0)	9 (26.5)
35–44	36 (32.1)	17 (32.7)	10 (29.4)
45–54	26 (23.2)	10 (19.2)	9 (26.5)
55–64	18 (16.1)	9 (17.3)	5 (14.7)
65 and over	4 (3.6)	2 (3.8)	1 (2.9)
Role			
Doctor: Family Physician	65 (58.0)	25 (48.2)	14 (41.0)
Doctor: Hospital Doctor	13 (11.7)	6 (11.5)	3 (8.8)
Doctor: Other	3 (2.7)	2 (3.8)	2 (6.0)
Doctor: Clinician Researcher	12 (10.7)	5 (9.6)	3 (8.8)
Nurse: Hospital Nurse	3 (2.7)	4 (7.7)	4 (11.8)
Nurse: Community Nurse	2 (1.8)	0 (0.0)	0 (0.0)
Nurse: Other	6 (5.4)	5 (9.6)	4 (11.8)
Other Healthcare Worker	8 (7.1)	5 (9.6)	4 (11.8)
Years of experience			
<5 years	22 (19.6)	11 (21.3)	7 (20.5)
5–10 years	24 (21.5)	7 (13.4)	2 (6.0)
>10 years	66 (58.9)	34 (65.3)	25 (73.5)
Additional respiratory qualifications or special interest			
Yes	72 (64.3)	35 (67.3)	21 (62.0)
No	40 (35.7)	17 (32.7)	13 (38.0)
Work setting			
Hospital	26 (23.2)	15 (29.0)	11 (32.4)
Primary care/ community	74 (66.1)	29 (55.7)	16 (47.1)
Other	12 (10.7)	8 (15.3)	7 (20.5)
Region			
Africa	14 (12.5)	5 (9.7)	4 (11.8)
Asia	37 (33.0)	21 (40.4)	12 (35.3)
Europe	46 (41.1)	18 (34.6)	12 (35.3)
North America	3 (2.7)	2 (3.8)	1 (2.9)
Oceania	3 (2.7)	2 (3.8)	1 (2.9)
South America	9 (8.0)	4 (7.7)	4 (11.8)
Country classification^a^			
High income	45 (40.2)	23 (44.2)	15 (44.1)
Upper-middle income	34 (30.4)	12 (23.0)	10 (29.4)
Lower-middle income	24 (21.4)	14 (27.0)	7 (20.5)
Low income	(8.0)	3 (5.8)	2 (6.0)

^a^Source: World Bank Country Classifications by income level: 2018–2019^26^.

Women accounted for 58% (*n* = 65) of the participants, and most (*n* = 90, 80.3%) were between the ages of 25–54 years. While some participants worked in hospital settings (also treating community patients) (*n* = 16, 14.3%), the majority worked mainly in primary care or community settings (*n* = 74, 66.1%). Overall, 65 (58%) were family physicians, 13 (11.6%) hospital doctors, 12 (10.7%), clinician-researchers, 11 (9.9%) were nurses and 8 (7.1%) were other healthcare workers. Sixty-six (58.9%) participants reported being in their roles for more than ten years, and 72 (64.3%) had respiratory-related special interests or qualifications. Rounds 2 and 3 included 52 and 34 of the original respondents respectively with a generally similar demographic distribution to round 1 except in round 3 where a larger proportion of women remained than in previous rounds (*n* = 22, (65%) women), a lower proportion of family physicians (*n* = 14, (41%)), but a greater proportion with more experience (*n* = 25 (73%)) reported 10 or more years’ experience in their role). The income distribution of countries was generally similar throughout the 3 rounds.

### Responses to the initial survey (round 1)

#### Question 1 (most common conditions)

Asthma was the most frequently mentioned respiratory condition encountered by respondents in their clinical practice (17.2%), followed by COPD (15.2%). However, as a clustered group of conditions, respiratory infections (TB, pneumonia, URTI, bronchitis/bronchiolitis, influenza) were mentioned most often (34.8% of all responses). Respiratory symptoms such as cough and breathlessness rather than specific clinical conditions were mentioned in 6.7% of responses.

#### Question 2 (most important conditions)

**Fig. 2:**
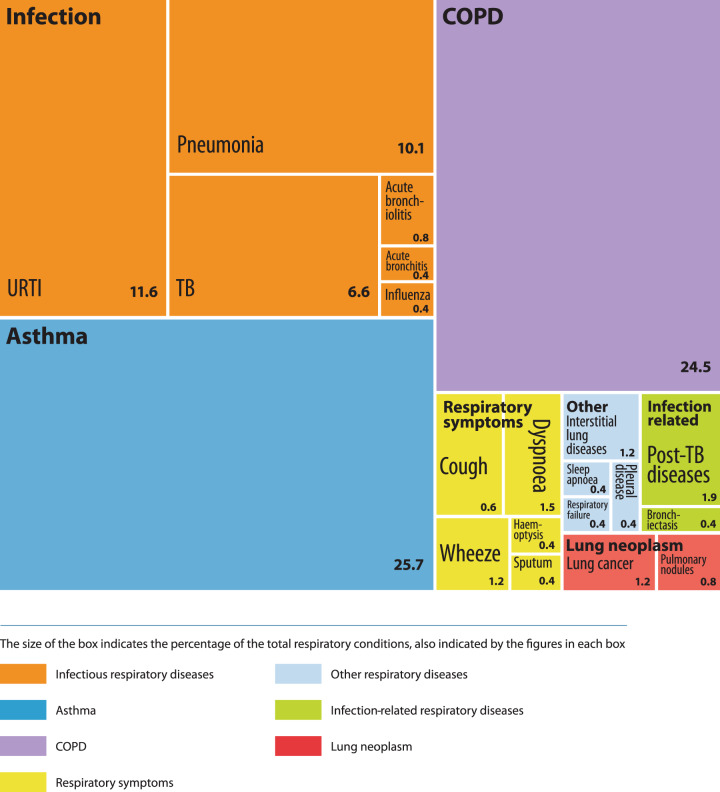
Proportional distribution of respiratory conditions identified by participants as most clinically important.

Although respiratory infections as a clustered group of conditions were perceived to be the most clinically important (29.9%), asthma was reported to be the most important single condition (25.7%), followed by COPD (24.5%), Fig. 2 illustrates the proportional distribution (percentages) of the most clinically important respiratory conditions.

#### Suggested research questions (question 3) and the Evidence Verification Stage

**Fig. 3:**
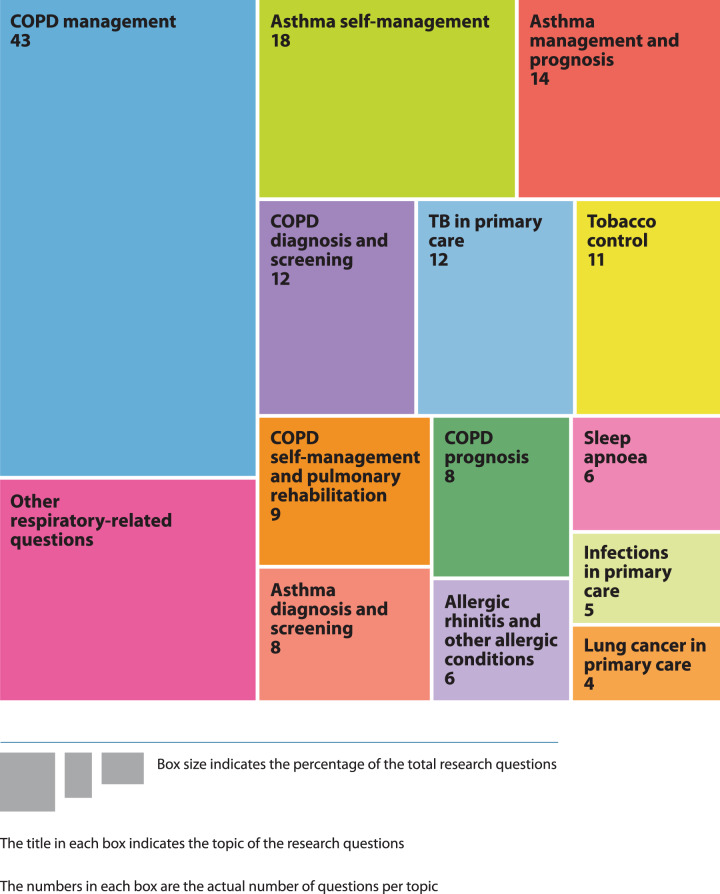
Proportional distribution of final research questions across the 14 topics after the Evidence Verification Stage.

A total of 608 research questions were suggested by participants and grouped into 19 topics representing common categories of respiratory conditions. After verification and review by the expert group, and removal of duplicates, 176 research questions were finalised, categorised pragmatically into 14 topics and entered into the remaining two e-Delphi rounds. Figure 3 illustrates the proportional distribution (frequencies) of respiratory research questions as finalised by experts in the Evidence Verification Stage. The greatest proportion of questions was related to the management of COPD, followed by asthma self-management, asthma management, COPD diagnosis and screening, tuberculosis in primary care and tobacco control.

### Consensus and ranking

Overall, 80% consensus was reached in 49 (27.7%) of the 176 rated questions. Asthma accounted for 19 (38.8%) of these questions while COPD accounted for 17 (34.7%) questions. Two questions (4%) reached a consensus of 100%: “What is the best way to manage chronic/ persistent cough in primary care?” and “What are the best ways to monitor asthma in primary care?”. Furthermore, 20 (40%) questions reached 90–99% consensus, while 27 questions (55.1%) reached a consensus of 80–89%. Table 2 lists the top 10 questions by consensus scores. Detailed rankings within asthma, COPD and other respiratory conditions are provided in Tables 3 to 5. Supplementary Table 3 lists all 176 questions with their scores.

**Table 2:** Top 10 primary care research respiratory priorities.

Question	Category	Consensus (%)^a^	Mean rating 0–5
What is the best way to manage chronic/ persistent cough in primary care?	Chronic/ persistent cough management	100	4.71
What are the best ways to monitor asthma in primary care?	Asthma monitoring	100	4.44
What steps could be taken to prevent exacerbations and progression of asthma?	Asthma management	97.1	4.38
How can brief advice be used more effectively to increase motivation to quit, and what elements are most efficient for a busy primary care practitioner?	Tobacco Control management	97.1	4.38
How should we best manage COPD in patients with cardiovascular diseases, arrhythmias and uncontrolled hypertension?	COPD management	97	4.35
What are the most effective strategies for ensuring sustained good inhaler techniques among asthma patients?	Asthma self-management	94.2	4.38
What methods could be used to enhance adherence to asthma controller therapy?	Asthma management	94.1	4.5
How could we improve COPD ‘patients’ adherence to inhalers? Which are the best methods to teach about inhaler use and how can we incorporate them in daily clinical practice?	COPD self-management	94.1	4.5
What is the best way to engage people with asthma in self-management?	Asthma self-management	94.1	4.44
How can we best educate healthcare professionals to improve the early recognition and diagnosis of COPD?	COPD diagnosis	94.1	4.44

Questions rated on a Likert scale (0: not important, –5: very important).

^a^% rating 4 (important) or 5 (very important).

**Table 3:** Consensus on the research priorities in asthma.

	Rank	Question	Consensus (%)^a^	Mean rating
*Asthma*				
Diagnosis	1	How could asthma be diagnosed earlier in primary care?	88.3	4.26
	2	How could asthma be diagnosed in settings with limited availability of diagnostic tests?	85.3	4.38
	3	What practical algorithms could distinguish between recurrent wheeze/ asthma and other acute respiratory diseases for young children?	85.3	4.24
Management	1	What steps could be taken to prevent exacerbations and progression of asthma?	97.1	4.38
	2	What methods could be used to enhance adherence to asthma controller therapy?	94.1	4.5
	3	What is the most effective management for acute exacerbation of asthma in children?	91.1	4.29
	4	How could guidelines be adapted to manage asthma in Lower-Middle-Income Countries (LMICs)?	88.2	4.35
	5	What is the role of intermittent therapy, such as SABA, ICS/SABA and ICS/LABA, in the management of asthma?	88.2	4.26
	6	When and how should asthmatic patients be stepped down from ICS?	85.3	4.09
	7	What is the best way to select drug therapy in children with asthma?	82.3	4.12
Monitoring	1	What are the best ways to monitor asthma in primary care?	100	4.44
	2	What are the best clinical tools to monitor asthmatic and allergic children in primary care in LMICs?	82.3	4.18
Self-management	1	What are the most effective strategies for ensuring sustained good inhaler techniques among asthma patients?	94.2	4.38
	2-a	What is the best way to engage people with asthma in self-management?	94.1	4.44
	2-b	What is the best way to support patients to improve their adherence to asthma medications?	94.1	4.44
	4	What are the best ways for healthcare professionals to engage patients in supported self-management and empower them to take control of their asthma?	94.1	4.24
	5	What are ‘physicians’ barriers to supporting patients to effectively self-manage their asthma in low-resource settings?	88.3	4.15
	6	What educational interventions are effective and cost-effective for children /families with asthma?	88.2	4.21
	7	What strategies/adaptations can help empower people with limited health literacy to effectively self-manage their asthma?	85.3	4.09

Questions rated on a Likert scale (0: not important, –5: very important).

*SABA* short-acting inhaled beta-agonists, *ICS* inhaled corticosteroids, *LABA* long-acting beta-agonists.

^a^% rating 4 (important) or 5 (very important).

**Table 4:** Consensus on the research priorities in COPD.

	Rank	Question	Consensus (%)^a^	Mean rating
*COPD*				
Diagnosis	1	How can we best educate healthcare professionals to improve the early recognition and diagnosis of COPD?	94.1	4.44
	2	How should we best diagnose COPD in settings where good quality spirometry is not available or not affordable?	91.2	4.32
	3	What are the most cost-effective and efficient approaches for identifying COPD, especially in low-resource settings?	88.3	4.26
	4	How effective are public awareness/education campaigns to improve awareness and earlier diagnosis of COPD?	82.3	4.26
Management	1	How should we best manage COPD in patients with cardiovascular diseases, arrhythmias and uncontrolled hypertension?	97.0	4.35
	2	How to tailor the current COPD management guidelines to suit those with comorbidities?	94.1	4.38
	3	How can we manage COPD patients with comorbidities in primary care using a personalised approach to reduce adverse reactions and limit disease progression?	91.2	4.38
	4	What is the optimal strategy for identifying and treating COPD exacerbations in primary care?	91.2	4.35
	5	Does shared care between primary care physicians and specialists improve the management of COPD patients and reduce exacerbations?	88.3	4.21
	6	How best could COPD treatments be tailored to suit different COPD phenotypes?	88.3	4.15
	7	How should COPD be managed in low- and middle-income countries, including rural community settings?	88.2	4.18
	8	How do primary care clinicians use spirometry findings to inform the ongoing management of COPD?	85.3	4.03
Monitoring	1	How do primary care clinicians use measures of disease progression in COPD to inform the care they provide? What is the impact of using measures of disease progression on quality of care and clinical outcomes?	88.3	4.15
Self-management	1	How could we improve ‘patients’ adherence to inhalers? Which are the best methods to teach about inhaler use and how can we incorporate them in daily clinical practice?	94.1	4.5
	2	How cost-effective are e-Health interventions, mobile and online applications (including wearables) in self-monitoring, symptoms control and adherence to medications in patients with COPD?	91.2	4.29
	3	What are the best engaging and supporting strategies for healthcare professionals to help improve self-management of COPD?	88.2	4.24
Prognosis	1	Is the early identification of COPD beneficial to patients in the long term?	85.3	4.32

Questions rated on a Likert scale (0, not important –5, very important).

^a^% rating 4 (important) or 5 (very important).

**Table 5:** Consensus on the research priorities in other respiratory conditions.

	Topic	Rank	Question	Consensus (%)^a^	Mean rating
*Other respiratory conditions*					
Diagnosis	TB	1	What are the best methods to increase detection of tuberculosis cases in primary healthcare or at the community level?	91.2	4.21
	Allergic rhinitis and other allergic conditions	2	What tools could help the primary care clinician differentiate between allergic and non-allergic rhinitis, rhinosinusitis, common cold and other clinically similar conditions?	88.2	4.24
	Infections in primary care	3	What are the best tools to help in triaging patients with respiratory infections to guide the use of antibiotics in community settings?	85.3	4.24
	Lung cancer in primary care	4-a	What is the best diagnostic algorithm for lung cancer for helping primary care doctors identify those at increased risk?	85.3	4.15
	Sleep apnoea	4-b	What is the best-validated screening tool for sleep-related breathing disorders, especially Obstructive Sleep Apnoea in the primary care setting?	85.3	4.15
Management	Other respiratory-related questions	1	What is the best way to manage chronic/ persistent cough in primary care?	100	4.71
	Tobacco control	2	How can brief advice be used more effectively to increase motivation to quit, and what elements are most efficient for a busy primary care practitioner?	97.1	4.38
	Tobacco control	3	What combination of interventions (e.g. brief advice, cost-free medications, adjunct counselling) are most effective for increasing patient quit rates in primary care practice?	91.2	4.32
	Tobacco control	4	What are the most effective models (including primary healthcare or specialist smoking cessation teams) for providing smoking cessation support services in different cultural and/or socioeconomic settings?	91.2	4.26
	Tobacco control	5	How can primary care clinicians in different countries be made more aware of strategies to prevent smoking in young people and pregnant women?	88.3	4.15
Monitoring	Tobacco control	1	How effective is monitoring patients following a quit attempt? What questions or simple instruments could be used to assess the risk of relapse in primary care consultations?	91.2	4.21
Self-management	Other respiratory-related questions	1	What are the most effective strategies to improve self-management of chronic respiratory diseases in primary care?	88.2	4.24
	Other respiratory-related questions	2	What are the most effective strategies to improve shared decisions and adherence when managing chronic lung diseases in primary care?	82.3	4.03

Questions rated on a Likert scale (0, not important –5, very important).

^a^% rating 4 (important) or 5 (very important).

### Qualitative analysis of cross-cutting themes from the initial questionnaire

**Fig. 4:**
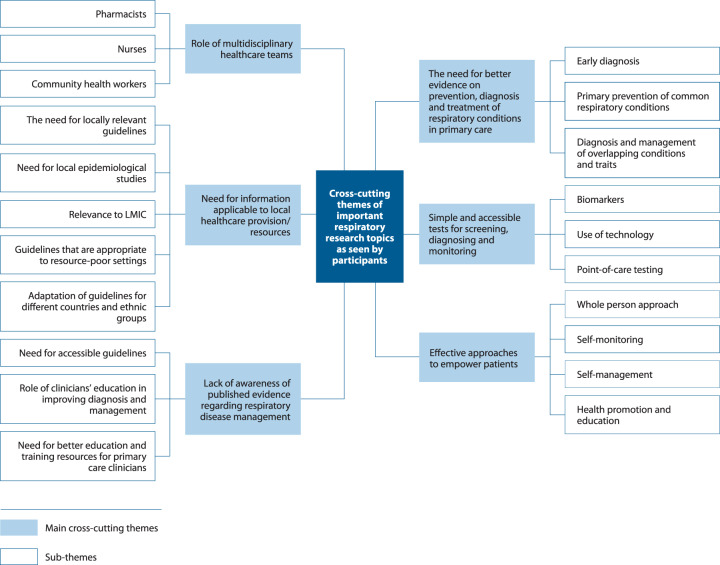
Summary of the 6 themes and sub-themes from the qualitative analysis of research questions.

A thematic analysis of the original 608 questions contributed by participants produced six cross-cutting themes relevant to primary care clinicians (Table 6) (Fig. 4). Despite the availability of relevant evidence, the first main theme highlights a need for education and accessible guidelines tailored for the primary care context reflecting a lack of awareness by some primary care clinicians of current recommendations about how to manage respiratory conditions. The second main theme provides insight into gaps in evidence for diagnosing and treating respiratory conditions in primary care. Themes 3 and 4 focus on the need for locally relevant information, both in terms of local evidence to inform decisions but also locally relevant and practical solutions for primary care, and particularly in low-resource settings. The final two areas of interest were the need to improve patient empowerment to manage their own conditions and the growing importance of the wider multidisciplinary healthcare team.

**Table 6:** Cross-cutting themes from qualitative analysis of open-ended round 1 questions.

Theme	Comments	Example of question received
Lack of awareness of published evidence regarding respiratory disease management	Many participants demonstrated a lack of knowledge of the available evidence regarding screening, diagnosing, and managing respiratory conditions in primary care	*“What is the best way to diagnose Asthma?”*
The need for better evidence on prevention, diagnosis and treatment of respiratory conditions in primary care	Some questions suggested a genuine gap in evidence and guidelines relevant to specific topics	*“What is the role of spirometry in the diagnosis of asthma at different age groups?“*
Need for information applicable to local healthcare provision/resources	Participants indicated a need for evidence, guidelines and epidemiological studies that directly related to their local populations.	*“What are the best feasible and effective asthma management guidelines that are appropriate for resource-poor settings?“*
Simple and accessible tests for screening, diagnosing and monitoring	A large proportion of suggested research questions demonstrated a need to explore or develop tests that are simple and feasible to perform in primary care to diagnose or manage respiratory conditions	“*How could point-of-care testing be used effectively in screening for COPD?“*
Effective approaches to empower patients	There was a significant emphasis on the need to explore tools and methods that could be used in primary care to empower patients with respiratory conditions in managing their own conditions.	*“What are the best self-management strategies for patients with chronic cough?“*
Role of multidisciplinary healthcare teams	Participants expressed interest in exploring the role of various healthcare professionals in the diagnosis, monitoring and management of respiratory conditions in primary care.	“*What is the role of community pharmacists in improving the prognosis of COPD patients?“*

## Discussion

We have used the e-Delphi method to rank the global priorities for respiratory research in primary care by drawing on the views and experiences of primary healthcare professionals from a wide range of settings and backgrounds. This supersedes the previous 2012 IPCRG priorities^14^ and uniquely provides a primary care perspective on respiratory problems.

While respiratory infections as a clustered group of conditions remained the most frequent and clinically important reason for consultation in primary care, COPD and asthma were considered the most frequent and clinically important individual conditions. This reflects both our previous findings^14^ and the current global burden^18^. TB was highlighted more frequently than in the 2012 IPCRG priority exercise, reflecting greater involvement of clinicians from LMICs.

While the most common research questions suggested by participants related to the diagnosis, management and self-management of COPD and asthma, there were a significant number of questions relating to tobacco control, reflecting a worldwide lack of progress in this area, especially in LMIC countries^10,19^. However, after the consensus-building stages of the e-Delphi, the most highly ranked research priorities (Table 2) concerned the management of chronic cough, brief advice for smoking cessation, management of multimorbidity, adherence to inhalers, monitoring of asthma and earlier diagnosis of COPD. The findings reflect the wide range of problems encountered in primary care, from prevention through to management of complexity, and emphasise the need for influencing behaviour change among both patients and clinicians. Many questions also related to how best to implement known effective interventions.

Additional cross-cutting themes included questions involving the role of the multidisciplinary team, the need for locally relevant data and guidance, empowerment of patients to be involved in their own healthcare, and the use of simple accessible tests for diagnosis and monitoring. A further theme identified the need for more effective clinical education for delivering best-practice care.

Compared with our previous prioritisation exercise^14^, there were a greater number of participants, fewer with an academic focus, and more from LMIC settings. In addition, open-ended questions were sought from a wider range of healthcare professionals without restriction on broad topic areas. This approach was reflected in our findings, with a more diverse range of research questions and the inclusion of additional topics e.g. TB, sleep apnoea and lung cancer. New topics emerged as priorities, such as the need for research about shared and multidisciplinary care, and the need for greater understanding of the role of inhaled corticosteroids in the management of COPD and asthma. However, some research topics remained important including the need for simple and accessible tools and tests, improvement of patient self-management skills (including inhaler adherence), and the most efficient and effective ways to help people quit tobacco in busy primary care settings. Research questions about comorbidities have progressed from simple descriptions to the management of people with multimorbidity. Training and education of primary care professionals remains an important topic.

Since the last IPCRG research prioritisation exercise, there have also been a number of other relevant respiratory research prioritisation exercises^20–23^ although all are more narrow in scope, focussing on specific conditions or geographical settings. The exercise specific to Portugal was based on the previous IPCRG research needs and set in primary care^20^. Similar to our recent findings, they emphasised the importance of methods of empowering patient self-management, optimising adherence to asthma and COPD medication and inhaler technique and reducing inappropriate antibiotic prescribing^20^. The patient-led EARIP asthma programme also prioritised optimising self-management support and medication adherence/inhaler technique but also highlighted the need for simple diagnostic tools and relevant training for healthcare professionals^21^. Another project-focused prioritisation publication considered specifically the research needs in LMIC countries in South Asia, prioritising research questions relevant to COPD awareness and early identification^22^. The James Lind alliance projects are more specific, and the current COPD exacerbation project is yet to be report^24^.

A major strength of this study is the large sample size and diverse representation of the participants from high-, middle- and low-income countries. While most participants in this study were primary care physicians, there was a representation of other healthcare professionals, including secondary care doctors with relevant experience, nurses, pharmacists, academic clinicians and other healthcare workers. Not surprisingly, two-thirds of participants reported an additional respiratory qualification or special interest in respiratory care, which may have affected generalisability, but only a few had a special interest in research.

While we received a large response rate for the initial survey of potential research questions, not all were able to participate in the subsequent rating rounds, in part due to the COVID-19 pandemic. Relatively fewer family physicians and participants with special respiratory interests participated in the rating rounds, which may have affected the final priority order. However, the distribution of participants from low, medium and high-income settings remained similar throughout.

One of the objectives of this study was to cover the breadth of respiratory conditions relevant to primary care. The bottom-up approach adopted with the open-ended questions helped to identify all important conditions observed in practice, including TB, lung cancer, interstitial lung diseases and sleep apnoea. A thorough evidence review stage was added to ensure that questions were refined and validated against current evidence by academic subject experts, thus avoiding duplication, questions already researched, or those not feasible for research. Inevitably there may have been some subjectivity involved; however, they were given standard guidance with a structured proforma and several experts were involved in most topic areas. The list of research questions was then prioritised by the non-expert participants, thus ensuring wider views were obtained on the most important questions.

Furthermore, using qualitative methods to analyse participants’ responses in-depth enabled us to triangulate the e-Delphi prioritisation and introduced an additional perspective on respiratory research gaps. This helped to highlight important issues beyond specific respiratory conditions that could be helpful in improving the care of respiratory patients in primary care globally.

Unfortunately, due to limited time and resources, it was not feasible to involve members of the public and patient groups or other stakeholders within this study. Their views should now be sought to provide further insight and alternative perspectives and we welcome Brief Communications and Comment.

Finally, full generalisability cannot be ensured, as we were only able to accept participants who had access to the internet and could complete the survey in English (including through self-arranged translators), which could be more of a problem in the primary care setting compared with secondary care.

The implications of our prioritised research questions will be far-reaching. It is widely accepted that the only way to achieve the United Nations Sustainable Development Goals, including a reduction in tobacco use, reduction in premature mortality from chronic respiratory diseases and improving wellbeing, is by orienting health systems towards primary care and supporting universal access^25^. However, this access needs to be to good quality primary healthcare^26^. Therefore, this study has identified clear knowledge gaps for primary care, which need to be addressed and tailored to the preferences of local primary care professionals.

Questions and themes elicited from this study can now be used to guide researchers and funders when planning research and allocating resources. Respiratory research has hitherto been relatively poorly funded, but it is clear from our work that more funding is urgently required and focussed on the greatest need. Our prioritised list of research questions generated by practising healthcare professionals ensures relevance and improves the chance of effective implementation. Research inspired by these priorities will contribute to the improvement in the respiratory health of patients in primary care, both locally and globally.

The requirement for prioritised respiratory research needs relevant to primary care is vital now more than ever^10^. The COVID-19 pandemic has led to unprecedented pressure on healthcare systems globally and prompted a re-organisation of the healthcare landscape^11^. This has impacted the way primary healthcare including vaccination for, diagnosis and management of respiratory conditions, is being delivered worldwide, with the use of remote and telephone consultations increased substantially^12^. COVID-19 has introduced a significant impact on the core competencies of primary care, which is affecting the continuity of care and changing the way primary healthcare will be provided in the near and distant future^14^. As COVID-19 has created new opportunities and innovations in medical research^15,16^, it will be important to tailor prioritised primary care respiratory research needs to fit into this new era of medical research. It is crucial, therefore, to allow for any new and dynamic changes in primary care when shaping prioritised primary care respiratory research.

The findings of this study also suggest a need to invest in evidence implementation with the publication of locally relevant primary care guidance, supported by effective methods of translation into practice. This exercise has also been signposted to areas of training needs for primary care professionals.

Finally, addressing these key areas of research will have wider implications for primary care because many of the respiratory research needs are generalisable to other conditions.

In conclusion, this e-Delphi exercise provides a prioritised list of respiratory-related research questions, which can be used by funders and researchers to commission and conduct research studies relevant to primary care clinicians globally. The findings also emphasise the need for primary care relevant guidance supported by effective approaches to achieve implementation. By driving this research agenda, we anticipate a shift in research funding and activity to improve the respiratory health and healthcare of patients managed in primary care worldwide.

## Supplementary information

Supplementary tables

REPORTING SUMMARY

Research prioritisation slideset redacted

## Data Availability

The data that support the findings of this study are available from the corresponding author upon reasonable request.
