# Supplementary material for: Prioritising primary care respiratory research needs: results from the 2020 International Primary Care Respiratory Group (IPCRG) global e-Delphi exercise
Source: NPJ Prim Care Respir Med. 2022 Jan 28;32:6. doi: 10.1038/s41533-021-00266-4 (PMC8799668; doi:10.1038/s41533-021-00266-4)
Supplement: Supplementary file 1 — Supplementary tables [file 41533_2021_266_MOESM1_ESM.docx]

**Supplementary Information**

**Supplementary tables 1,2 and 3**

**Prioritising primary care respiratory research needs: results from the 2020 International Primary Care Respiratory Group (IPCRG) global e-Delphi exercise**

| TB (in primary care)  ***Supplementary Table 1:* List of experts who contributed to the Evidence Verification Stage by topic** | Bruce Kirenga | Uganda |
| --- | --- | --- |
| Condition | Expert names | Country |
| Asthma diagnosis and screening | Jaime Correia de Sousa | Portugal |
|  | Karin Lisspers | Sweden |
| Asthma management and prognosis | Amanda Barnard | Australia |
|  | Björn Ställberg | Sweden |
|  | Stanley J. Szefler | USA |
| Children with asthma | Mercedes Escarrer | Spain |
|  | Louis Moral | Spain |
| Asthma self-management | Hilary Pinnock | UK |
|  | Steph Taylor | UK |
| COPD diagnosis and screening | Rachel Jordan | UK |
|  | Andy Dickens | UK |
| COPD management | Alice Turner | UK |
|  | Shamil Haroon | UK |
|  | Ioanna Tsiligianni | Greece |
|  | Job FM van Boven | Netherlands |
| Multi-morbidity | Ioanna Tsiligianni | Greece |
| COPD self-management/pulmonary rehabilitation | Sally Singh | UK |
|  | Niels Chavannes | Netherlands |
| COPD prognosis | Professor Peymané Adab | UK |
|  | Rachel Jordan | UK |
| Infections/ pneumonia (in primary care) | Cliodna McNulty | UK |
| Allergic rhinitis/ other allergic conditions | Dermot Ryan | UK |
|  | Mercedes Escarrer | Spain |
|  | Louis Moral | Spain |
| Lung cancer (in primary care) | David Weller | UK |
|  | Alex Kayongo | Uganda |
| Tobacco control | Daniel Kotz | Germany |
|  | Sophia Papadakis | Greece |
| Air pollution | Niels Chavannes | Netherlands |
|  | Alice Turner | UK |
| Health economics: | Job FM van Boven | Netherlands |
| Sleep Apnoea | Izolde Bouloukaki | Greece |
| Lung Fibrosis/ ILD/ occupational lung diseases | David Price | UK |
| Other respiratory conditions/ general | Björn Ställberg | Sweden |
|  | Karin Lisspers | Sweden |
|  | Jaimie Correia de Sousa | Portugal |

***Supplementary Table 1:* Distribution of participants and countries based on income classification***

| High-Income | Number of Participants | Upper Middle-Income | Number of Participants | Lower Middle-Income | Number of Participants | Low -Income | Number of Participants |
| --- | --- | --- | --- | --- | --- | --- | --- |
| Australia | 3 | Malaysia | 5 | India | 2 | Ethiopia | 4 |
| Belgium | 2 | Brazil | 6 | Bangladesh | 6 | Mozambique | 1 |
| Canada | 3 | China | 8 | Kyrgyzstan | 11 | Uganda | 4 |
| Chile | 3 | Romania | 10 | Angola | 2 |  |  |
| France | 1 | Turkey | 5 | Cape Verde | 3 |  |  |
| Germany | 1 |  |  |  |  |  |  |
| Greece | 5 |  |  |  |  |  |  |
| Ireland | 1 |  |  |  |  |  |  |
| Macao | 5 |  |  |  |  |  |  |
| Netherlands | 4 |  |  |  |  |  |  |
| Norway | 2 |  |  |  |  |  |  |
| Portugal | 7 |  |  |  |  |  |  |
| Sweden | 2 |  |  |  |  |  |  |
| United Kingdom | 6 |  |  |  |  |  |  |
| Number of participants from High-income countries:  n (%) | 45 (40.2) | Number of participants from Upper middle-income countries:  n (%) | 34 (30.4) | Number of participants from Lower middle-income countries:  n (%) | 24 (21.4) | Number of participants from low-income countries:  n (%) | 9 (8) |
| Number of high-income countries:  n (%) | 14 (52) | Number of upper middle-income countries:  n (%) | 5 (18.5) | Number of lower middle-income countries:  n (%) | 5 (18.5) | Number of low-income countries:  n (%) | 3 (11) |
|  |  |  | | Number of participants from LMIC:  n (%) | | 67 (59.8) | |
|  |  |  | | Number of LMIC countries:  n (%) | | 13 (48) | |

** Source: World Bank Country Classifications by income level: 2018-2019* ^17^*.*

***Supplementary Table 3***  Full list of the 176 respiratory research questions finalised by experts in the Evidence Verification stage

| Topic | Question | Consensus score | Mean Rank |
| --- | --- | --- | --- |
| Asthma diagnosis and screening | How could asthma be diagnosed earlier in primary care? | 88.3 | 4.26 |
| 8 questions | What practical algorithms could distinguish between recurrent wheeze/ asthma and other acute respiratory diseases for young children? | 85.3 | 4.24 |
|  | How could asthma be diagnosed in settings with limited availability of diagnostic tests? | 85.3 | 4.38 |
|  | What is the role of spirometry in the diagnosis of asthma at different age groups? | 79.4 | 4.15 |
|  | What is the best way to diagnose exercise-induced asthma in primary care? | 61.8 | 3.65 |
|  | What tools could be used in community pharmacies to assess the probability of asthma? | 41.2 | 3.32 |
|  | How could the implementation of pocket guidelines help the diagnosis of asthma? | 44.1 | 3.53 |
|  | How could we achieve an international consensus on the definition of asthma? | 76.5 | 4.06 |
| Asthma management and prognosis | What is the role of intermittent therapy, such as SABA, ICS/SABA and ICS/LABA, in the management of asthma? (SABA=Short-acting inhaled beta-agonists, ICS=Inhaled Corticosteroids, LABA=Long-acting beta-agonists) | 88.2 | 4.26 |
| 14 questions | When and how should asthmatic patients be stepped down from ICS? | 85.3 | 4.09 |
|  | What is the most effective management for acute exacerbation of asthma in children? | 91.1 | 4.29 |
|  | What is the best way to select drug therapy in children with asthma? | 82.3 | 4.12 |
|  | What are the best ways to manage post-viral asthma? | 52.9 | 3.62 |
|  | Are there any new non-inhaled asthma medications that are effective in the treatment of asthma? | 41.2 | 3.53 |
|  | Are there any new cost-effective care models that are effective in managing asthma in a primary care setting? | 70.6 | 3.91 |
|  | How could biomarkers be used in clinical practice for managing asthma? | 47.1 | 3.56 |
|  | What methods could be used to enhance adherence to asthma controller therapy? | 94.1 | 4.5 |
|  | What are the best ways to monitor asthma in primary care? | 100 | 4.44 |
|  | What steps could be taken to prevent exacerbations and progression of asthma? | 97.1 | 4.38 |
|  | How could guidelines be adapted to manage asthma in Lower- Middle- Income Countries (LMIC)? | 88.2 | 4.35 |
|  | What are the best clinical tools to monitor asthmatic and allergic children in primary care in LMIC? | 82.3 | 4.18 |
|  | What is the importance of different clinical phenotypes in the prognosis of asthma with early onset in childhood? | 67.7 | 4.06 |
| Asthma self-management | What educational interventions are effective and cost-effective for children /families with asthma? | 88.2 | 4.21 |
| 18 questions | What is the best way to support patients to improve their adherence to asthma medications? | 94.1 | 4.44 |
|  | What are the most effective strategies for ensuring sustained good inhaler techniques among asthma patients? | 94.2 | 4.38 |
|  | What is the best way to engage people with asthma in self-management? | 94.1 | 4.44 |
|  | What self-monitoring techniques are appropriate for people with mild intermittent asthma? | 73.6 | 3.97 |
|  | What is the best way to support accurate recognition among asthma patients of worsening of signs and symptoms (e.g. questionnaires and novel biomarkers)? | 70.6 | 3.88 |
|  | What strategies/adaptations can help empower people with limited health literacy to effectively self-manage their asthma? | 85.3 | 4.09 |
|  | Would universal, international, validated, shared, self-management plans help in improving the management of patients with asthma? | 52.9 | 3.62 |
|  | Which patients are most likely to need support to self-manage? | 52.9 | 3.62 |
|  | What are 'patients' barriers to effectively self-managing their asthma in low resource settings? | 79.5 | 4.12 |
|  | What are 'physicians' barriers to supporting patients to effectively self-manage their asthma in low resource settings? | 88.3 | 4.15 |
|  | What are the best ways for health care professionals to engage patients in supported self-management and empower them to take control of their asthma? | 94.1 | 4.24 |
|  | What is the role of pharmacists in supporting asthma self-management? | 50 | 3.62 |
|  | What is the role of community health workers in supporting asthma self-management? | 61.7 | 3.76 |
|  | What is the potential for digital options in improving recognition of asthma deterioration? | 70.6 | 3.94 |
|  | Are digital options effective in improving adherence to medications and self-management among asthma patients compared to traditional modes of delivery? | 70.6 | 3.94 |
|  | What is the impact of over-the-counter availability on the use of asthma medications? | 53 | 3.68 |
|  | What are the best ways to discourage over-reliance on beta-agonists in patients with asthma? | 67.7 | 3.97 |
| COPD diagnosis and screening | Which are the best methods to identify Asthma-COPD Overlap Syndrome (ACOS) in primary care? | 73.5 | 3.94 |
| 12 questions | What are the most effective approaches for optimising uptake of invitations for COPD screening? | 70.6 | 3.82 |
|  | Is the early identification of COPD beneficial to patients in the long-term? | 85.3 | 4.32 |
|  | What are the most cost-effective and efficient approaches for identifying COPD, especially in low-resource settings? | 88.3 | 4.26 |
|  | How should we best diagnose COPD in settings where good quality spirometry is not available or not affordable? | 91.2 | 4.32 |
|  | Is it feasible to use microspirometry to assess lung function amongst those with poor coordination or low cognitive ability? | 55.8 | 3.56 |
|  | What are the clinical characteristics of patients diagnosed by microspirometry alone, compared with those diagnosed using quality diagnostic spirometry? | 52.9 | 3.68 |
|  | Is lung function measured by spirometry stable over time and is there a need for multiple measurements before diagnosis? | 50 | 3.59 |
|  | What is the burden and epidemiology of COPD in settings with no current information? | 50 | 3.59 |
|  | What are the reference values for countries with no current relevant information? | 58.9 | 3.82 |
|  | How can we best educate healthcare professionals to improve early recognition and diagnosis of COPD? | 94.1 | 4.44 |
|  | How effective are public awareness/education campaigns to improve awareness and earlier diagnosis of COPD? | 82.3 | 4.26 |
| COPD management | How to best manage patients with Asthma-COPD Overlap Syndrome (ACOS)? | 70.6 | 3.94 |
| 43 questions | How existing guidelines for asthma and COPD should be modified for the diagnosis and management of occupational Asthma-COPD Overlap Syndrome (ACOS)? | 58.8 | 3.71 |
|  | How should asthma-COPD overlap be monitored? | 70.5 | 3.82 |
|  | What are the most effective and cost-effective therapies for people with mild or case-found/screen-detected COPD? | 76.4 | 4 |
|  | What are the optimal care pathways for the management of people with screen-detected COPD? | 67.6 | 3.82 |
|  | Can we effectively manage different COPD types with appropriate treatments? | 88.3 | 4.15 |
|  | In end-stage COPD patients, what are the benefits/ risks of de-prescribing inhaled therapy? | 52.9 | 3.59 |
|  | What is the cost-benefit analysis of LABA/ICS in developing countries? | 58.9 | 3.65 |
|  | How can we best apply personalised approaches to tailor the most appropriate treatments to patients with COPD (triple therapy versus dual versus mono-therapy vs ICS/LABA, co-morbidities, etc.?) | 64.7 | 3.82 |
|  | When should ICS be stopped when they are already in use in COPD? | 79.4 | 4.15 |
|  | How do we individualise the decision of choosing an inhaler based on patients characteristics taking into consideration advantages, disadvantages and inhalers specific characteristics? | 70.6 | 3.94 |
|  | How could we improve 'patients' adherence to inhalers? Which are the best methods to teach about inhaler use and how can we incorporate them in daily clinical practice? | 94.1 | 4.5 |
|  | What is the optimal strategy for identifying and treating COPD exacerbations in primary care? | 91.2 | 4.35 |
|  | How should we best manage asymptomatic patients with emphysema on Computed tomography (CT)? | 38.3 | 3.35 |
|  | What are the factors leading to ICS overuse in the treatment of COPD in primary care? How could this be minimised? | 64.7 | 3.74 |
|  | Does shared care between primary care physicians and specialists improve the management of COPD patients and reduce exacerbations? | 88.3 | 4.21 |
|  | Are educational interventions for community health agents about care for patients and families of COPD patients cost-effective? | 70.6 | 3.97 |
|  | How effective are multidisciplinary approaches involving primary health care centres, community pharmacies and community centres in improving the clinical outcome for patients with COPD? | 64.7 | 3.85 |
|  | How frequently, and using which approach, should we monitor COPD patients in the community? Should this vary by severity? | 67.7 | 3.85 |
|  | What is the role of community pharmacists in improving the prognosis of COPD patients? | 38.2 | 3.35 |
|  | What are effective treatments for biomass-related COPD and how should biomass-related COPD be managed? | 53 | 3.65 |
|  | How should COPD be managed in low- and middle-income countries, including rural community settings? | 88.2 | 4.18 |
|  | Are micro-spirometers an adequate substitute for spirometry for monitoring the progression of COPD in primary care? | 67.6 | 3.85 |
|  | Can portable particulate monitors be used to prevent exacerbations and prevent deterioration of health for patients with COPD? | 52.9 | 3.59 |
|  | How effective and cost-effective is a point of care C-reactive protein (CRP) testing (or other biomarkers) for guiding therapy for preventing and treating exacerbations of COPD in primary care? | 55.9 | 3.59 |
|  | How effective are biomarkers in assessing adherence to COPD treatments? | 47.1 | 3.59 |
|  | How do primary care clinicians use spirometry findings to inform the ongoing management of COPD? | 85.3 | 4.03 |
|  | How do primary care clinicians use measures of disease progression in COPD to inform the care they provide? What is the impact of using measures of disease progression on quality of care and clinical outcomes? | 88.3 | 4.15 |
|  | What should be the referral criteria for secondary care for patients with COPD? | 73.5 | 3.91 |
|  | What is the prognostic value of eosinophils and other novel biomarkers in predicting exacerbations? | 58.8 | 3.71 |
|  | What is the role of eosinophils in the treatment effect of ICS? | 44.2 | 3.5 |
|  | How effective and how safe is the use of eosinophil level to step-down ICS treatment? | 58.9 | 3.68 |
|  | Can Fractional Exhaled nitric oxide (FeNo) be used in primary care to identify ICS-responsive patients? | 47.1 | 3.47 |
|  | How can we adapt care to patients with low health literacy? | 73.5 | 3.91 |
|  | What is the impact of financial restrictions on COPD management and adherence to medications? | 64.7 | 3.68 |
|  | How useful is the ABCD assessment tool for classifying and managing patients? Are there better ways to classify COPD patients? | 73.6 | 3.79 |
|  | How should we best manage COPD in patients with cardiovascular diseases, arrhythmias and uncontrolled hypertension? | 97 | 4.35 |
|  | How can we manage COPD patients with comorbidities in primary care using a personalised approach to reduce adverse reactions and limit disease progression? | 91.2 | 4.38 |
|  | How to tailor the current COPD management guidelines to suit those with comorbidities? | 94.1 | 4.38 |
|  | How best to improve awareness, early identification and screening of mental health disorders in patients with COPD, and does this improve outcomes? | 73.5 | 3.91 |
|  | What is the clinical- and cost-effectiveness of management plans that are implemented based on prognostic score categories, rather than routine care? | 52.9 | 3.62 |
|  | Could telephone counselling for patients with COPD help in managing their COPD? | 52.9 | 3.74 |
|  | Are digital applications cost-effective for the management of COPD in patients at high risk for exacerbations? | 61.8 | 3.79 |
| COPD self-management/pulmonary rehabilitation | What are the best engaging and supporting strategies for health care professionals to help improve self-management of COPD? | 88.2 | 4.24 |
| 9 questions | How cost-effective are e-Health interventions, mobile and online applications (including wearables) in self-monitoring, symptoms control and adherence to medications in patients with COPD? | 91.2 | 4.29 |
|  | How effective are individual self-management compared to group self-management sessions? | 53 | 3.62 |
|  | What are the most effective ways to deliver self-management plans to patients in settings with low numbers of doctors and health workers? | 79.4 | 3.88 |
|  | What are the patient-related or other barriers that prevent patients from completing pulmonary rehabilitation programmes? What interventions would help to reduce drop-out rates? | 64.7 | 3.82 |
|  | What are physician-related barriers to providing self-management support to patients? | 79.4 | 4.03 |
|  | What is the effectiveness of alternative forms of exercise (such as Tai Chi, dance etc.) among people with COPD, and what are the most active components of such interventions? | 35.3 | 3.24 |
|  | What is the effect of adapted pulmonary rehabilitation on clinical outcomes in low resource settings where pharmacotherapy is less affordable/accessible? | 79.4 | 4.03 |
|  | What is the feasibility and effectiveness of offering a choice of activities within a pulmonary rehabilitation programme, compared to traditional only activities, on the uptake and clinical outcomes? | 52.9 | 3.71 |
| COPD prognosis | What is the prognosis of case-found / screened COPD patients, compared to i) those who remain undetected till later in the disease process, and ii) COPD patients diagnosed through usual care? | 64.7 | 3.82 |
| 8 questions | What are the characteristics and prognosis of patients with normal spirometry but chronic respiratory symptoms and exacerbation-like events indicative of COPD? | 58.9 | 3.85 |
|  | What are the characteristics and prognosis of people with airflow obstruction who have never smoked, compared to ever smokers with or without airflow obstruction? | 53 | 3.71 |
|  | Can the performance of COPD risk prediction scores be improved by adding other factors such as clinical measures or assessment of exposure to risk factors? | 41.2 | 3.44 |
|  | To what extent are risk prediction scores stable if alternative measures are substituted within them (e.g. different measures of exercise capacity rather than Six-Minute Walk Test)? | 38.2 | 3.44 |
|  | What is the validity of existing prognostic scores in different populations, e.g. those with COPD in LMIC, or those with other conditions? | 52.9 | 3.59 |
|  | What thresholds or cut-offs should be used for existing prognostic scores, to allow patients to be categorised into clinically meaningful groups? | 44.2 | 3.5 |
|  | Does COPD caused by biomass fuel have the same phenotype and prognosis as COPD caused by smoking? | 44.1 | 3.53 |
| Infections in primary care | What are the best tools to help in triaging patients with respiratory infections to guide the use of antibiotics in community settings? | 85.3 | 4.24 |
| 5 questions | What is the optimal self-care treatment strategy for acute and chronic sinusitis? | 61.8 | 3.74 |
|  | How effective are self-management options for patients with cough and Upper Respiratory Tract Infections (URTI) in primary care? | 44.2 | 3.5 |
|  | What is the current understanding of the general public about the causes, illness course and complication rate, and how they can self-care in upper airways infections? | 47.1 | 3.44 |
|  | What factors are related to a failure of the flu vaccine response in patients with chronic lung disease? | 50 | 3.71 |
| Allergic rhinitis/ other allergic conditions | What tools could help the primary care clinician differentiate between allergic and non-allergic rhinitis, rhinosinusitis, common cold and other clinically similar conditions? | 88.2 | 4.24 |
| 6 questions | What are the best-stratified treatment options for rhinitis (both allergic and non-allergic) that suits variable severities and chronicity? | 79.4 | 3.97 |
|  | How best to monitor allergic rhinitis? Self-monitoring (applications) or clinical monitoring? Symptom-based or biomarker-based? | 70.6 | 3.79 |
|  | What is the best way to accurately diagnose rhinitis (allergic/non-allergic) in pre-school children (<5 y)? | 79.4 | 3.97 |
|  | How best to diagnose and manage rhinitis in low-income countries? | 73.5 | 3.85 |
|  | In children and adults with upper viral respiratory tract infection and allergic rhinitis, is the use of oral antihistamines and/or topical nasal steroids beneficial in reducing symptom severity and duration? | 61.7 | 3.79 |
| Lung cancer (in primary care) | What is the best diagnostic algorithm for lung cancer for helping primary care doctors identify those at increased risk? | 85.3 | 4.15 |
| 4 questions | What is the current, and potential future role, of biomarkers in early lung cancer detection? | 58.8 | 3.76 |
|  | How can primary care best engage with new treatments for lung cancer (such as immunotherapies), and take an active role in community-based management? | 35.3 | 3.41 |
|  | How can we ensure that early diagnosis and screening strategies for lung cancer do not increase health inequalities? | 73.5 | 3.85 |
| TB (in primary care) | What are the most useful specific host or pathogen-derived molecular and immunological markers of early tuberculosis (TB) infection? | 47.1 | 3.59 |
| 12 questions | What are the genetic correlates of TB susceptibility? | 17.7 | 3 |
|  | Is there a better test to detect early non-compliance or resistant cases compared to sputum acid-fast bacilli staining? | 50 | 3.62 |
|  | What are the most useful specific host or pathogen-derived molecular and immunological markers for extra-pulmonary tuberculosis? | 32.3 | 3.38 |
|  | What is the most effective chemotherapy for treating Latent tuberculosis infection (LTBI)? | 44.1 | 3.62 |
|  | What are the best methods to increase detection of tuberculosis cases in primary health care or at the community level? | 91.2 | 4.21 |
|  | What are the best ways to improve compliance with treatments in patients with TB? | 79.4 | 4.03 |
|  | What are the best ways to prevent exacerbation of post-TB bronchiectasis? | 52.9 | 3.76 |
|  | What are the most effective drugs with the fewest side effects for the treatment of Multidrug-resistant TB (TBMDR)? | 64.7 | 3.76 |
|  | Is Levofloxacin effective in treating adult close contacts of TB patients who are not immunocompromised? | 35.3 | 3.41 |
|  | What are the barriers and challenges of a TB control program for immigrants? | 64.7 | 3.88 |
|  | What are the clinical features that could help in predicting prognosis in patients with TB? | 58.8 | 3.76 |
| Tobacco control | How can brief advice be used more effectively to increase motivation to quit, and what elements are most efficient for a busy primary care practitioner? | 97.1 | 4.38 |
| 11 questions | What are the benefits of using questionnaires (e.g. 'willingness to 'quit', 'addiction to 'nicotine') in routine clinical practice? Which ones are the most accurate and useful? | 70.5 | 3.88 |
|  | How can primary care clinicians in different countries be made more aware of strategies to prevent smoking in young people and pregnant women? | 88.3 | 4.15 |
|  | What are the most effective models (including primary healthcare or specialist smoking cessation teams) for providing smoking cessation support services in different cultural and/or socioeconomic settings? | 91.2 | 4.26 |
|  | How effective is monitoring patients following a quit attempt? What questions or simple instruments could be used to assess the risk of relapse in primary care consultations? | 91.2 | 4.21 |
|  | What are the barriers and facilitators for patients' adherence to smoking cessations treatments? | 79.4 | 4.03 |
|  | What are primary care 'patients' knowledge and beliefs regarding the risk of tobacco use and the importance of smoking cessation? | 58.8 | 3.76 |
|  | What are the best practices for working with political leaders and business to increase support for smoking cessation? | 67.7 | 3.91 |
|  | What are the best practices for treating tobacco dependence in patients with depression? | 61.8 | 3.74 |
|  | What are the best practices for increasing primary healthcare 'professionals' knowledge regarding nicotine addiction and evidence-based tobacco treatment? | 64.7 | 3.79 |
|  | What combination of interventions (e.g. brief advice, cost-free medications, adjunct counselling) are most effective for increasing patient quit rates in primary care practice? | 91.2 | 4.32 |
| Sleep Apnoea | What is the best-validated screening tool for sleep-related breathing disorders, especially Obstructive Sleep Apnoea in the primary care setting? | 85.3 | 4.15 |
| 6 questions | What is the optimal management of patients with sleep-related breathing disorders in the primary care setting? | 76.5 | 3.79 |
|  | What are the best strategies to improve adherence to continuous positive airway pressure (CPAP) in the management of patients with sleep apnoea in primary care? | 64.7 | 3.71 |
|  | What are the best ways to monitor sleep-related breathing disorders in primary care? | 67.7 | 3.71 |
|  | What is the prognosis of patients with sleep-related breathing disorders who are using CPAP and have adequate follow up by a family physician? | 52.9 | 3.56 |
|  | What are the most effective strategies to educate family physicians about sleep-related breathing disorders? | 58.9 | 3.65 |
| Other respiratory-related questions | What is the best way to manage chronic/ persistent cough in primary care? | 100 | 4.71 |
| 20 questions | What are the most effective non-pharmacological methods of managing breathlessness? | 73.5 | 3.94 |
|  | Are there simple treatable traits or phenotypic algorithms which are more useful in classifying and managing patients with chronic respiratory disease in primary care? | 64.7 | 3.68 |
|  | What are the best ways to help patients with chronic respiratory disease to increase their exercise capacity? | 79.4 | 3.91 |
|  | What are the best strategies to involve other healthcare professionals in the management of chronic respiratory diseases in primary care? | 67.7 | 3.88 |
|  | What are the barriers and facilitators to early diagnosis, management and treatment of respiratory conditions in primary care? | 76.5 | 3.97 |
|  | How can community pharmacists be involved in the care of patients with chronic respiratory diseases? | 38.3 | 3.29 |
|  | How useful are Quality of Life questionnaires in the assessment of respiratory diseases? | 58.9 | 3.65 |
|  | Which tools should be used to identify patients' health beliefs and understanding of chronic respiratory diseases in primary care? | 73.5 | 3.85 |
|  | What are the most effective strategies to improve shared decision and adherence when managing chronic lung diseases in primary care? | 82.3 | 4.03 |
|  | What are the most effective strategies to improve self-management of chronic respiratory diseases in primary care? | 88.2 | 4.24 |
|  | How can information and communication technologies help in monitoring chronic respiratory diseases? | 79.4 | 4.03 |
|  | How could telemedicine strategies help to improve adherence to treatment in patients with chronic respiratory disease in LMIC? | 70.6 | 3.94 |
|  | How could e-Health be used in the treatment and monitoring of chronic lung disease? | 76.5 | 3.91 |
|  | Are digital options effective for providing access to healthcare or managing health service workload compared to traditional methods? | 55.9 | 3.56 |
|  | Which interventions reduce the consequences of outdoor and indoor air pollution on chronic lung disease? | 53 | 3.62 |
|  | What is the best way to identify those meriting investigations for Interstitial Lung Disease? | 41.2 | 3.47 |
|  | Does earlier diagnosis and intervention improve outcomes for Idiopathic pulmonary fibrosis? | 50 | 3.53 |
|  | What are the most effective treatment options to manage bronchiectasis in primary care? | 55.9 | 3.74 |
|  | How could we identify patients with bronchiectasis who will progress more rapidly and have a higher burden of the disease? | 70.5 | 3.79 |
