# Supplementary material for: Prioritising primary care respiratory research needs: results from the 2020 International Primary Care Respiratory Group (IPCRG) global e-Delphi exercise
Source: NPJ Prim Care Respir Med. 2022 Jan 28;32:6. doi: 10.1038/s41533-021-00266-4 (PMC8799668; doi:10.1038/s41533-021-00266-4)
Supplement: Supplementary file 3 — Research prioritisation slideset redacted [file 41533_2021_266_MOESM3_ESM.pptx]

## Slide 1
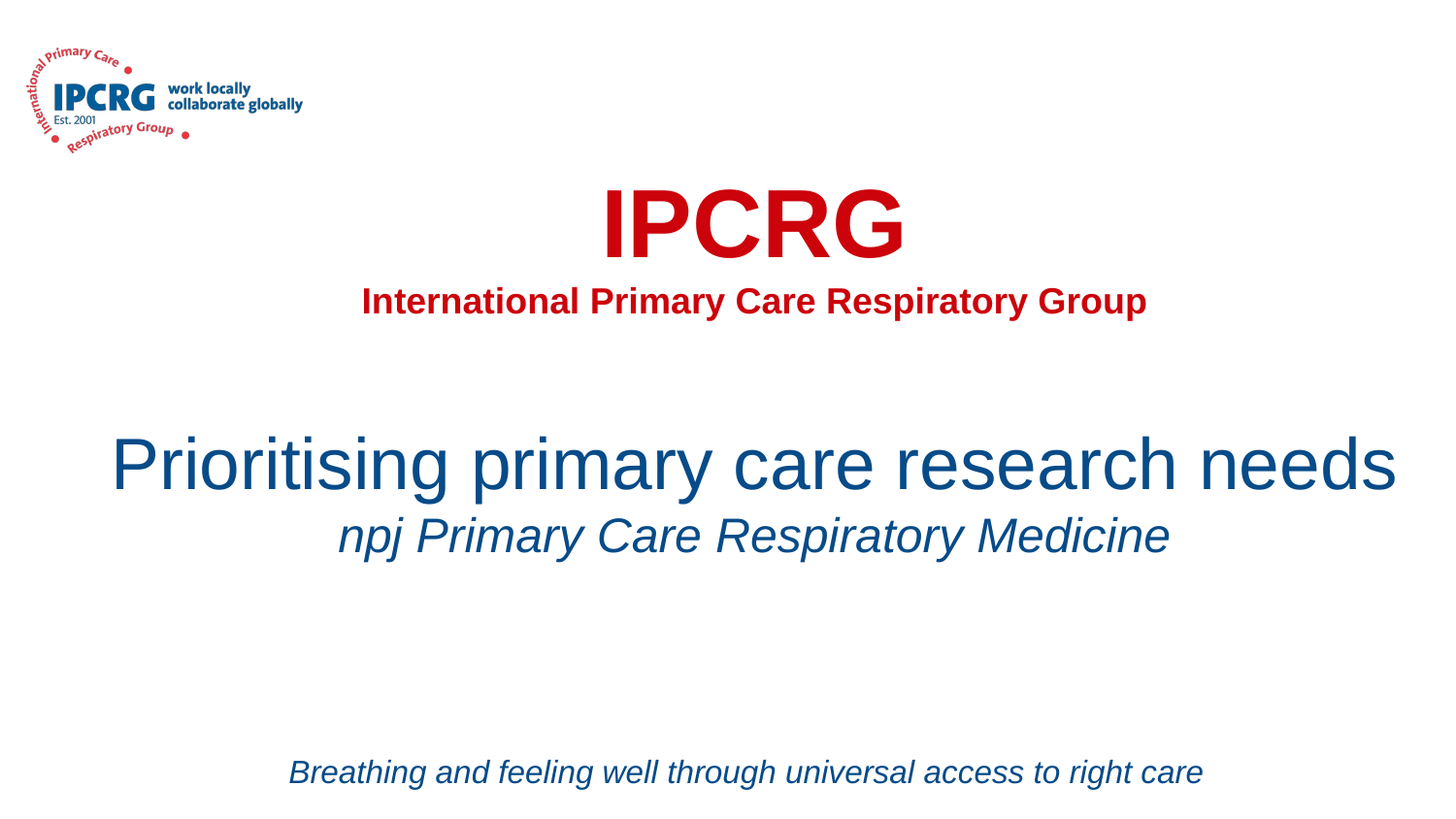

# IPCRG International Primary Care Respiratory Group
Prioritising primary care research needs
npj Primary Care Respiratory Medicine

## Slide 2
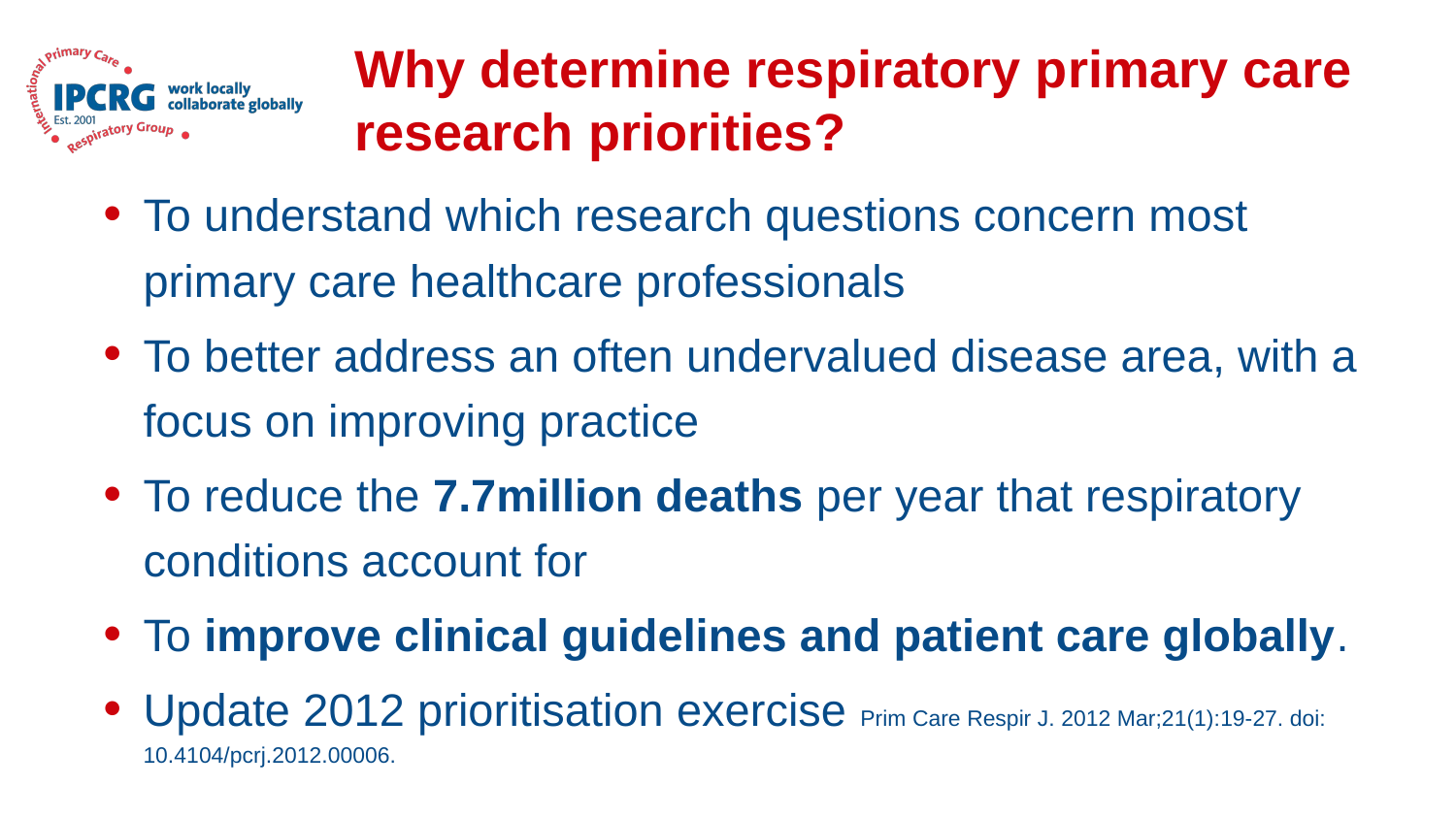

# Why determine respiratory primary care research priorities?
To understand which research questions concern most primary care healthcare professionals
To better address an often undervalued disease area, with a focus on improving practice
To reduce the 7.7million deaths per year that respiratory conditions account for
To improve clinical guidelines and patient care globally.
Update 2012 prioritisation exercise Prim Care Respir J. 2012 Mar;21(1):19-27. doi: 10.4104/pcrj.2012.00006.

## Slide 3
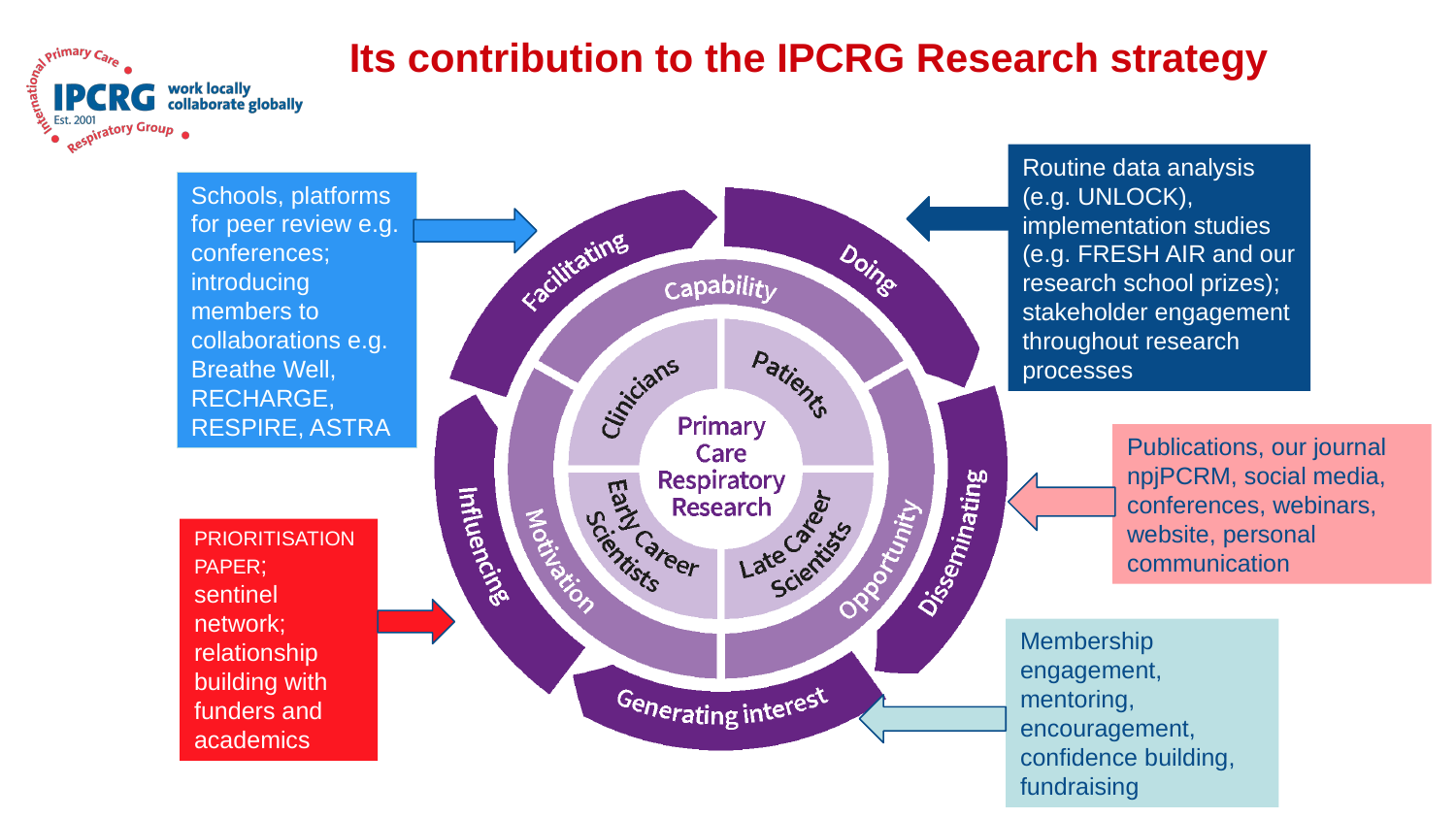

# Its contribution to the IPCRG Research strategy
Routine data analysis (e.g. UNLOCK), implementation studies (e.g. FRESH AIR and our research school prizes); stakeholder engagement throughout research processes
Schools, platforms for peer review e.g. conferences; introducing members to collaborations e.g. Breathe Well, RECHARGE, RESPIRE, ASTRA
Publications, our journal npjPCRM, social media, conferences, webinars, website, personal communication
PRIORITISATION PAPER; sentinel network; relationship building with funders and academics
Membership engagement, mentoring, encouragement, confidence building, fundraising

## Slide 4
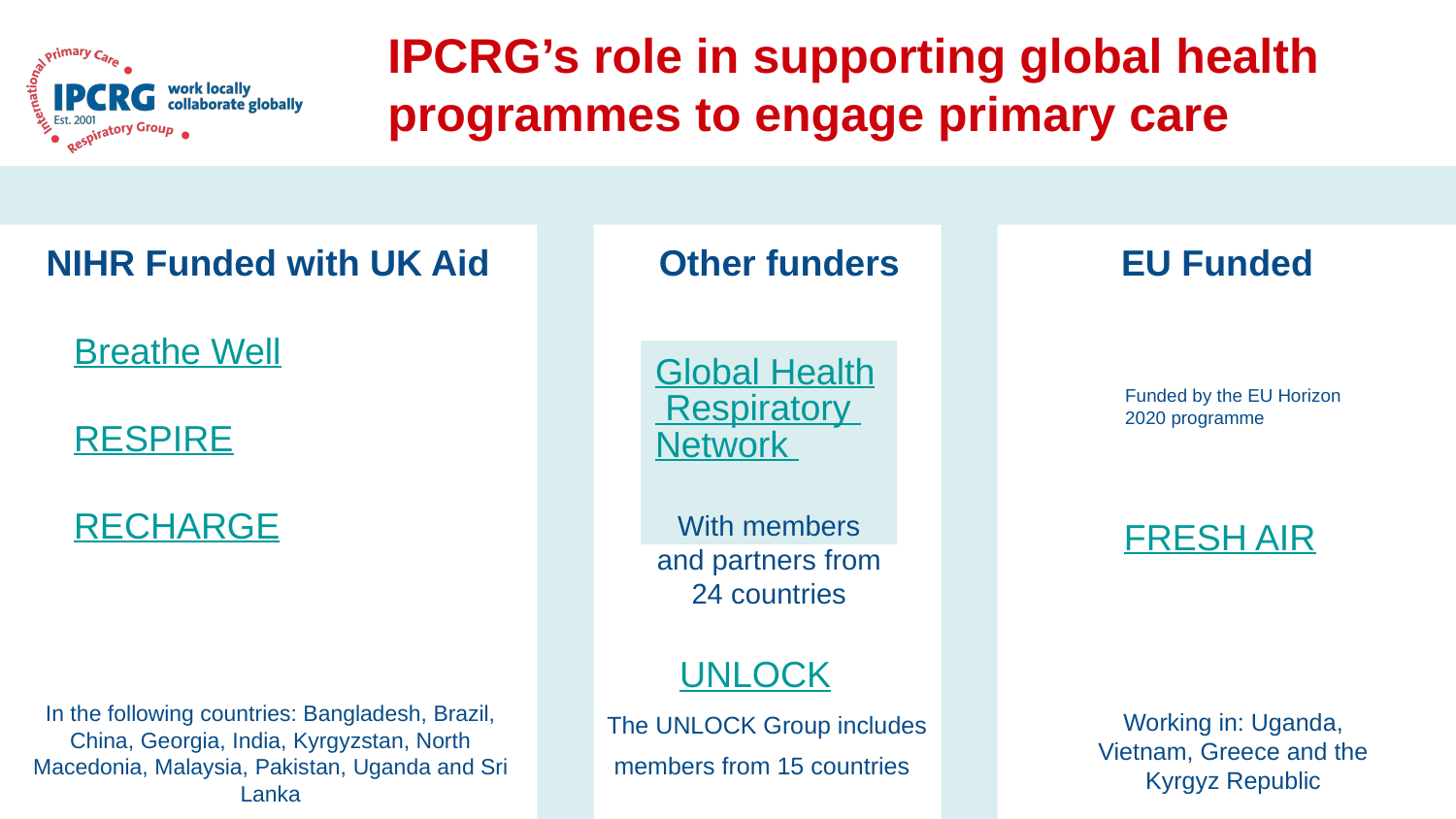

# IPCRG’s role in supporting global health programmes to engage primary care
NIHR Funded with UK Aid
Other funders
EU Funded
Breathe Well
RESPIRE
RECHARGE
Global Health Respiratory Network
With members and partners from 24 countries
Funded by the EU Horizon 2020 programme
FRESH AIR
UNLOCK
In the following countries: Bangladesh, Brazil, China, Georgia, India, Kyrgyzstan, North Macedonia, Malaysia, Pakistan, Uganda and Sri Lanka
Working in: Uganda, Vietnam, Greece and the Kyrgyz Republic
The UNLOCK Group includes members from 15 countries

## Slide 5
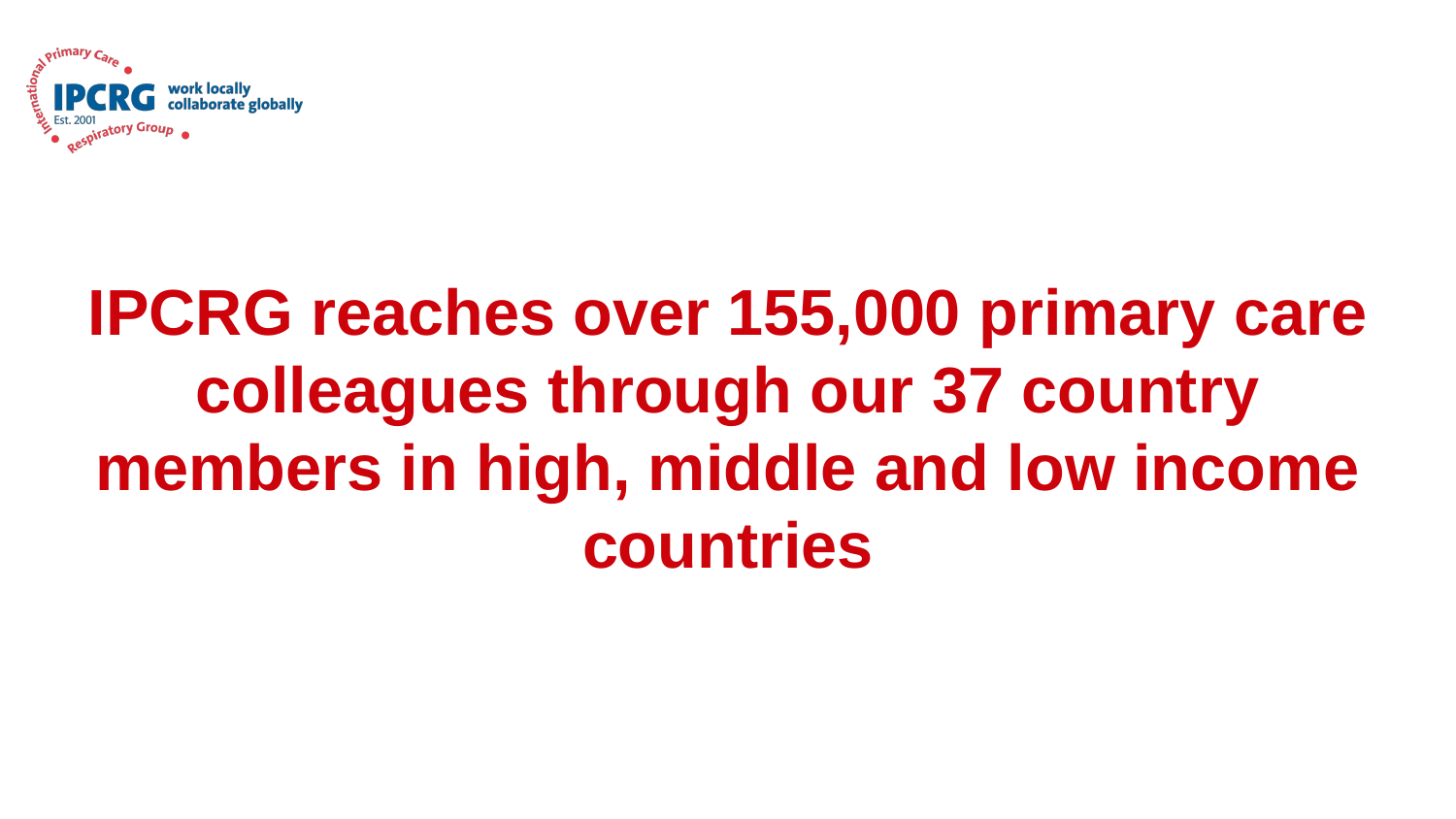

# IPCRG reaches over 155,000 primary care colleagues through our 37 country members in high, middle and low income countries

## Slide 6
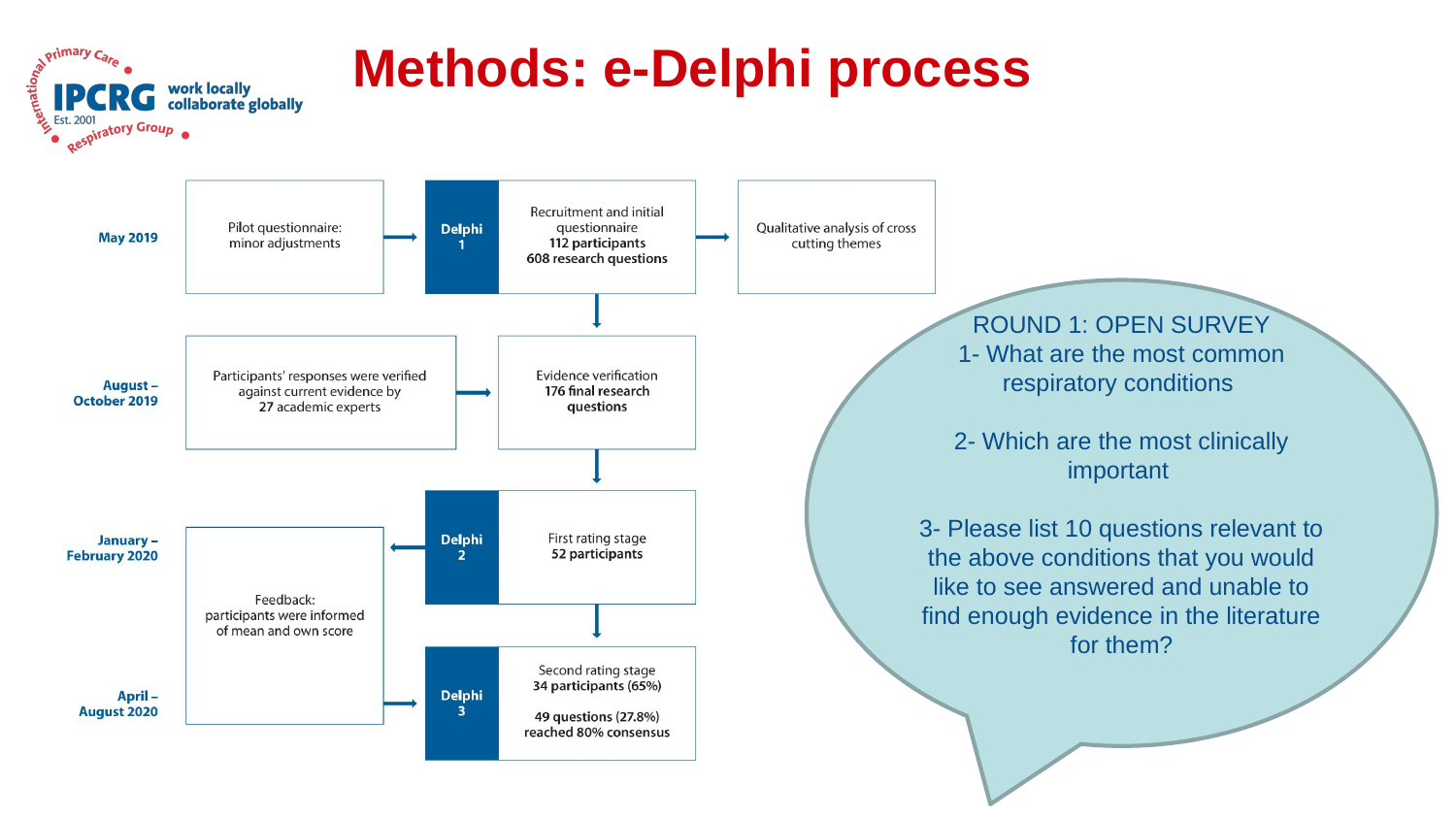

# Methods: e-Delphi process
ROUND 1: OPEN SURVEY
1- What are the most common respiratory conditions
2- Which are the most clinically important
3- Please list 10 questions relevant to the above conditions that you would like to see answered and unable to find enough evidence in the literature for them?

## Slide 7
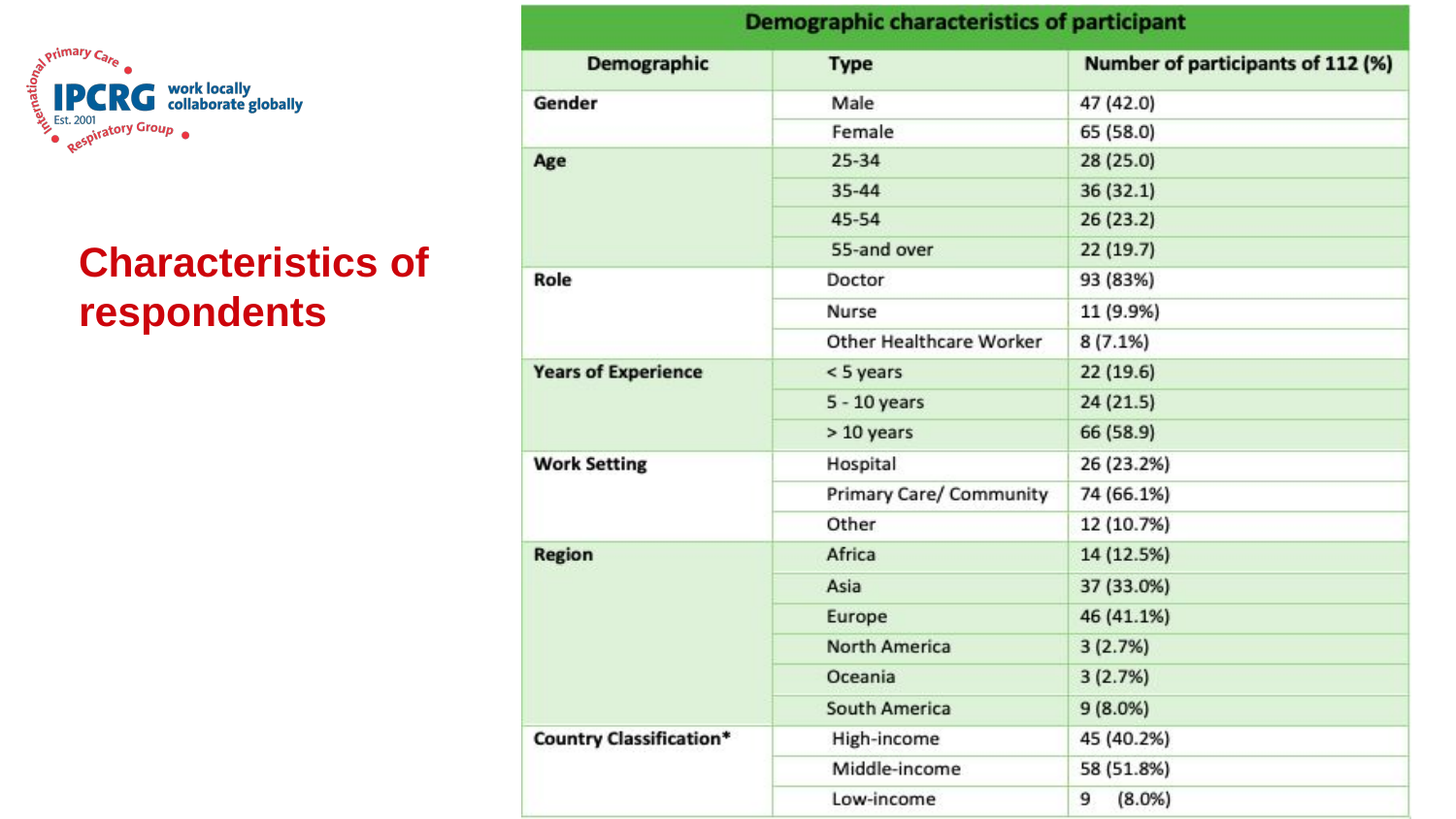

# Characteristics of respondents

## Slide 8
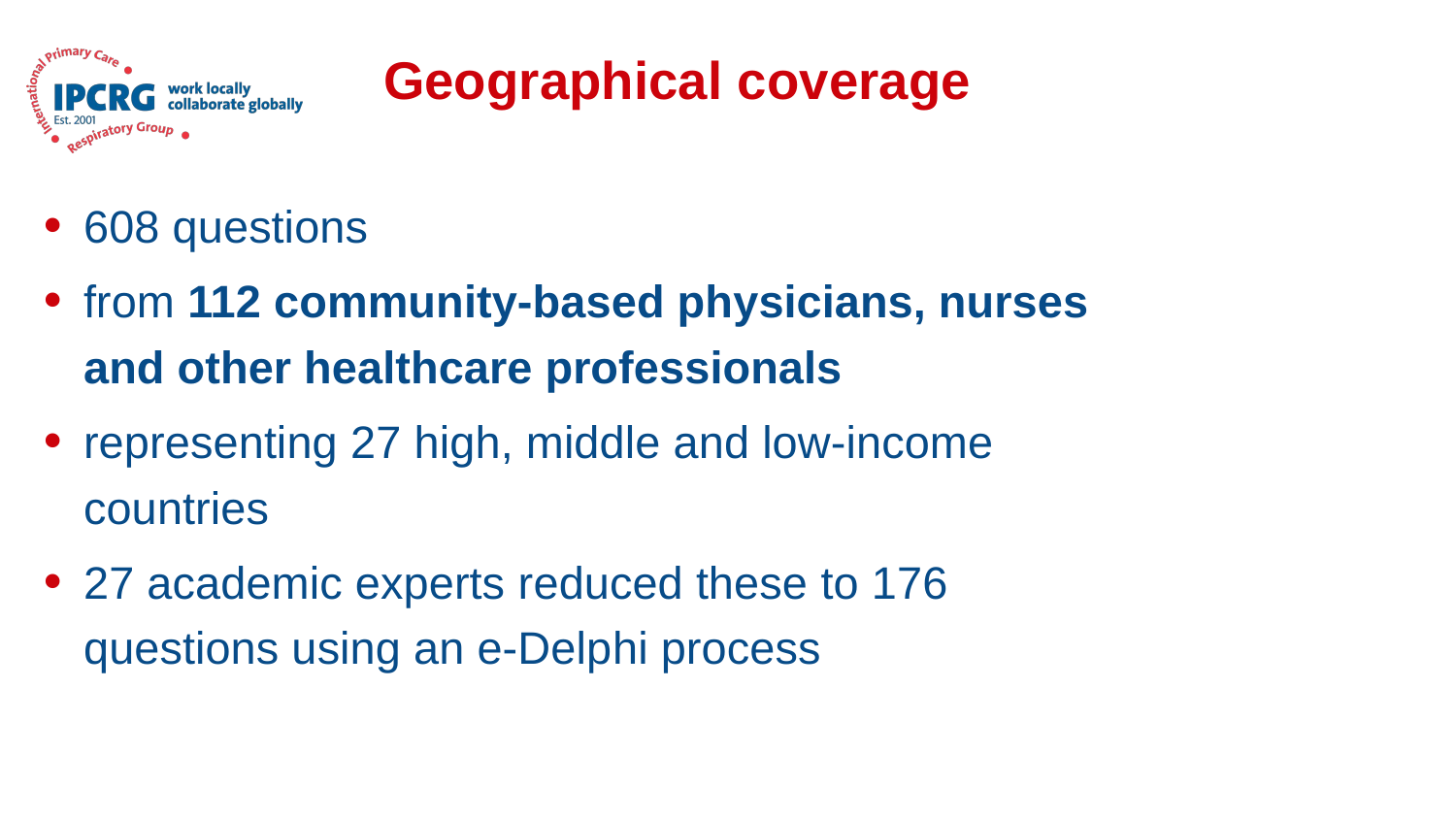

# Geographical coverage
608 questions
from 112 community-based physicians, nurses and other healthcare professionals
representing 27 high, middle and low-income countries
27 academic experts reduced these to 176 questions using an e-Delphi process

## Slide 9
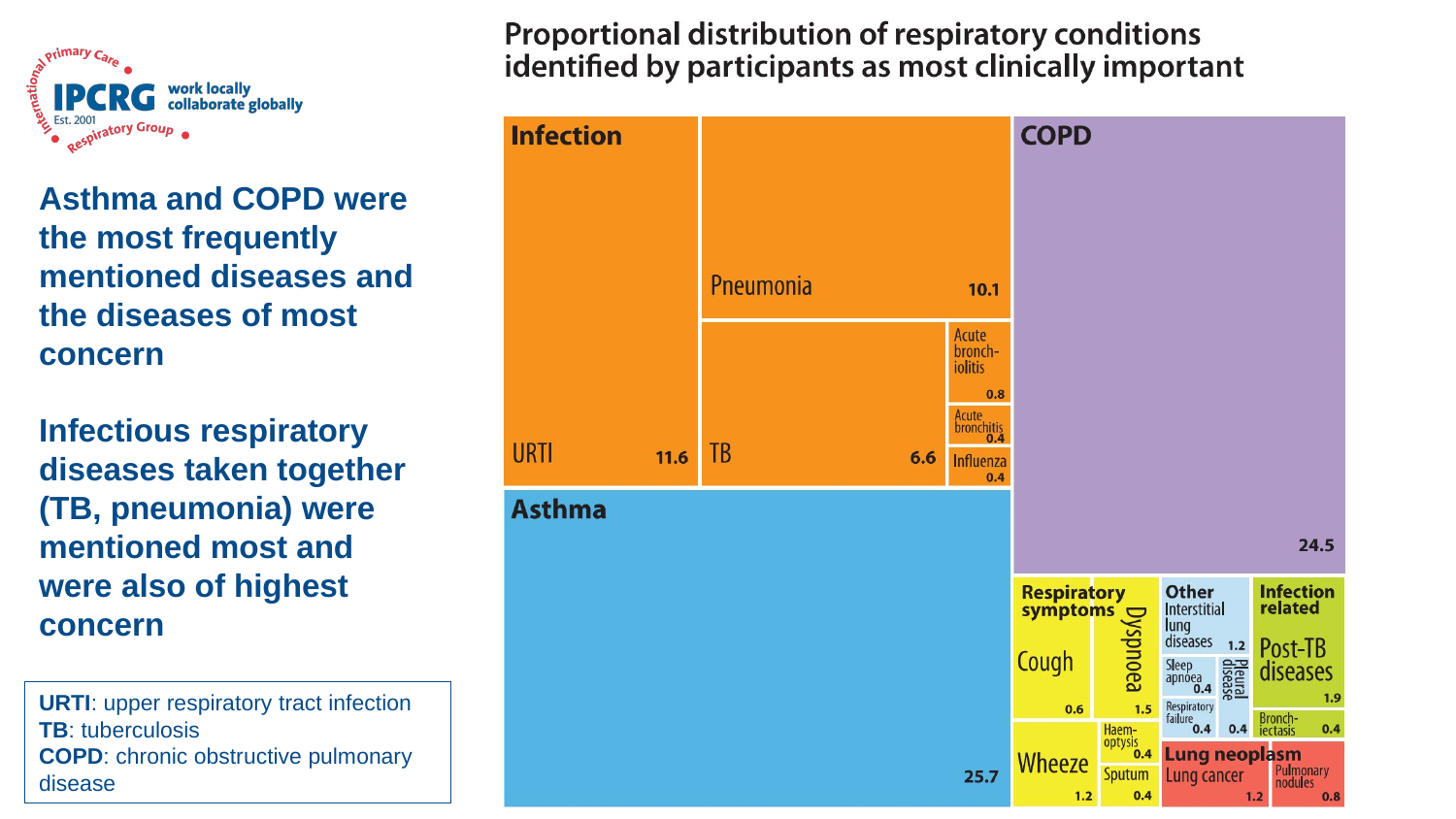

Asthma and COPD were the most frequently mentioned diseases and the diseases of most concern
Infectious respiratory diseases taken together (TB, pneumonia) were mentioned most and were also of highest concern
URTI: upper respiratory tract infection
TB: tuberculosis
COPD: chronic obstructive pulmonary disease

## Slide 10
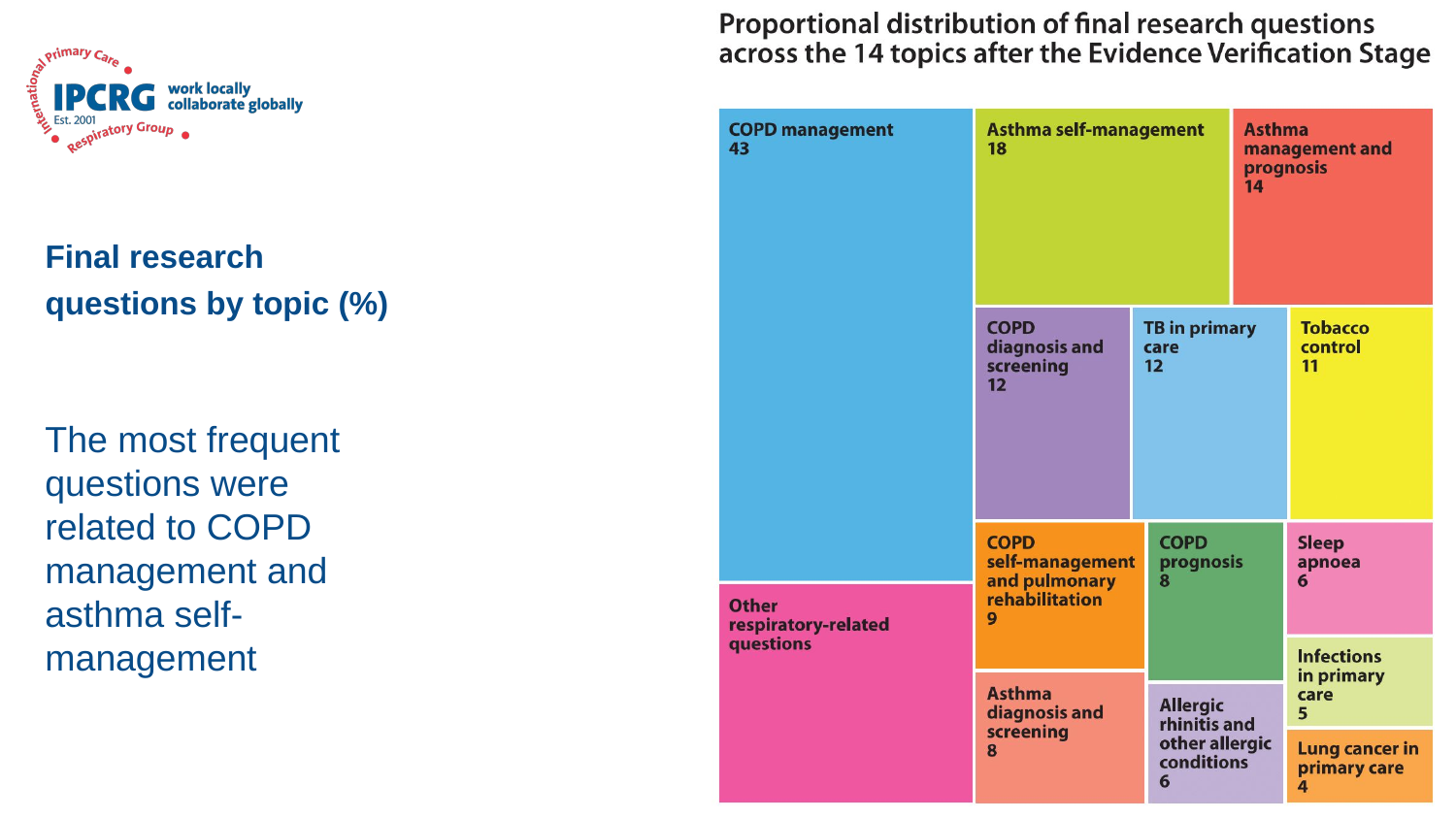

Final research questions by topic (%)
The most frequent questions were related to COPD management and asthma self-management

## Slide 11
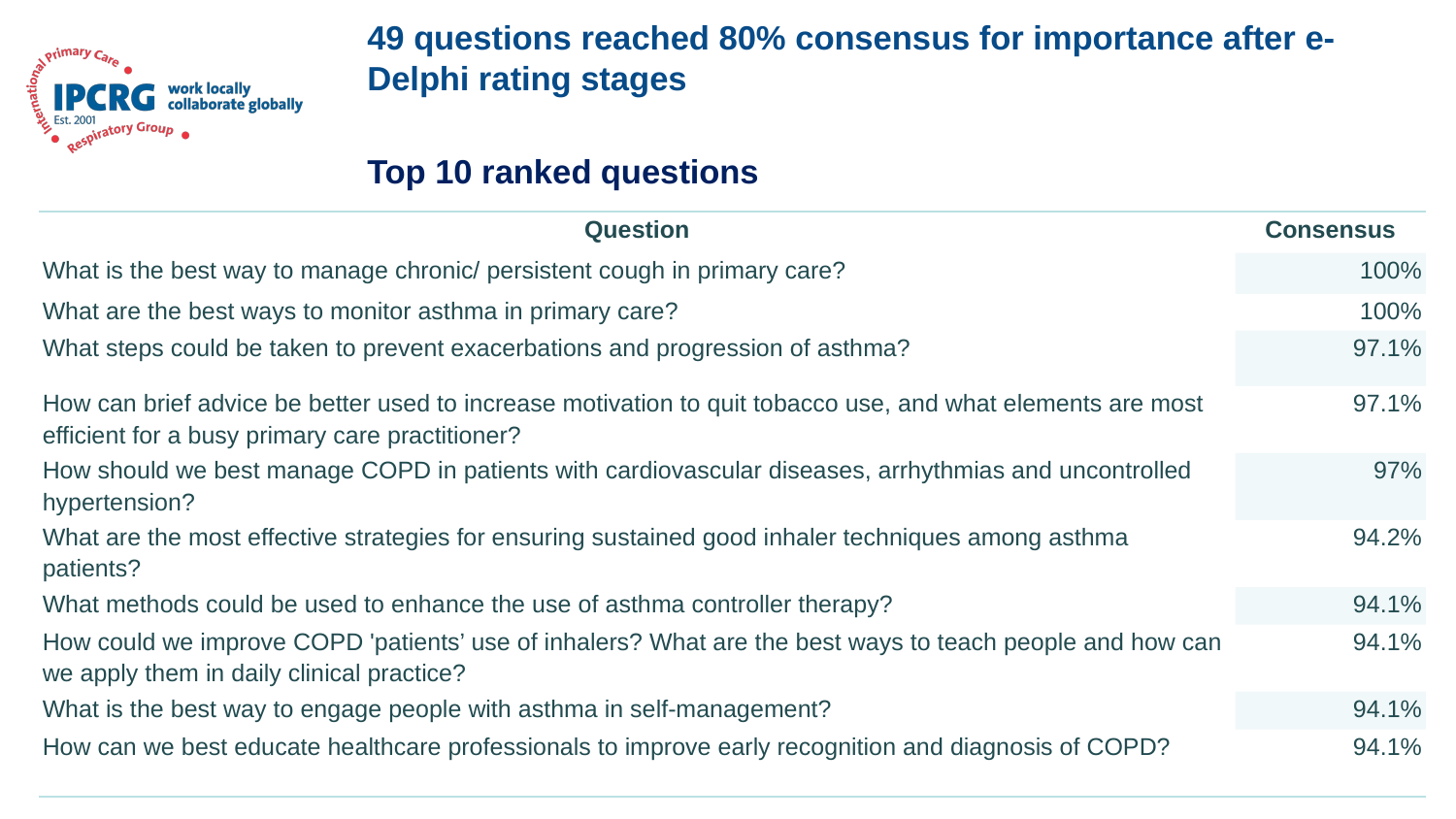

# 49 questions reached 80% consensus for importance after e-Delphi rating stages Top 10 ranked questions
| Question | Consensus |
| --- | --- |
| What is the best way to manage chronic/ persistent cough in primary care? | 100% |
| What are the best ways to monitor asthma in primary care? | 100% |
| What steps could be taken to prevent exacerbations and progression of asthma? | 97.1% |
| How can brief advice be better used to increase motivation to quit tobacco use, and what elements are most efficient for a busy primary care practitioner? | 97.1% |
| How should we best manage COPD in patients with cardiovascular diseases, arrhythmias and uncontrolled hypertension? | 97% |
| What are the most effective strategies for ensuring sustained good inhaler techniques among asthma patients? | 94.2% |
| What methods could be used to enhance the use of asthma controller therapy? | 94.1% |
| How could we improve COPD 'patients’ use of inhalers? What are the best ways to teach people and how can we apply them in daily clinical practice? | 94.1% |
| What is the best way to engage people with asthma in self-management? | 94.1% |
| How can we best educate healthcare professionals to improve early recognition and diagnosis of COPD? | 94.1% |

## Slide 12
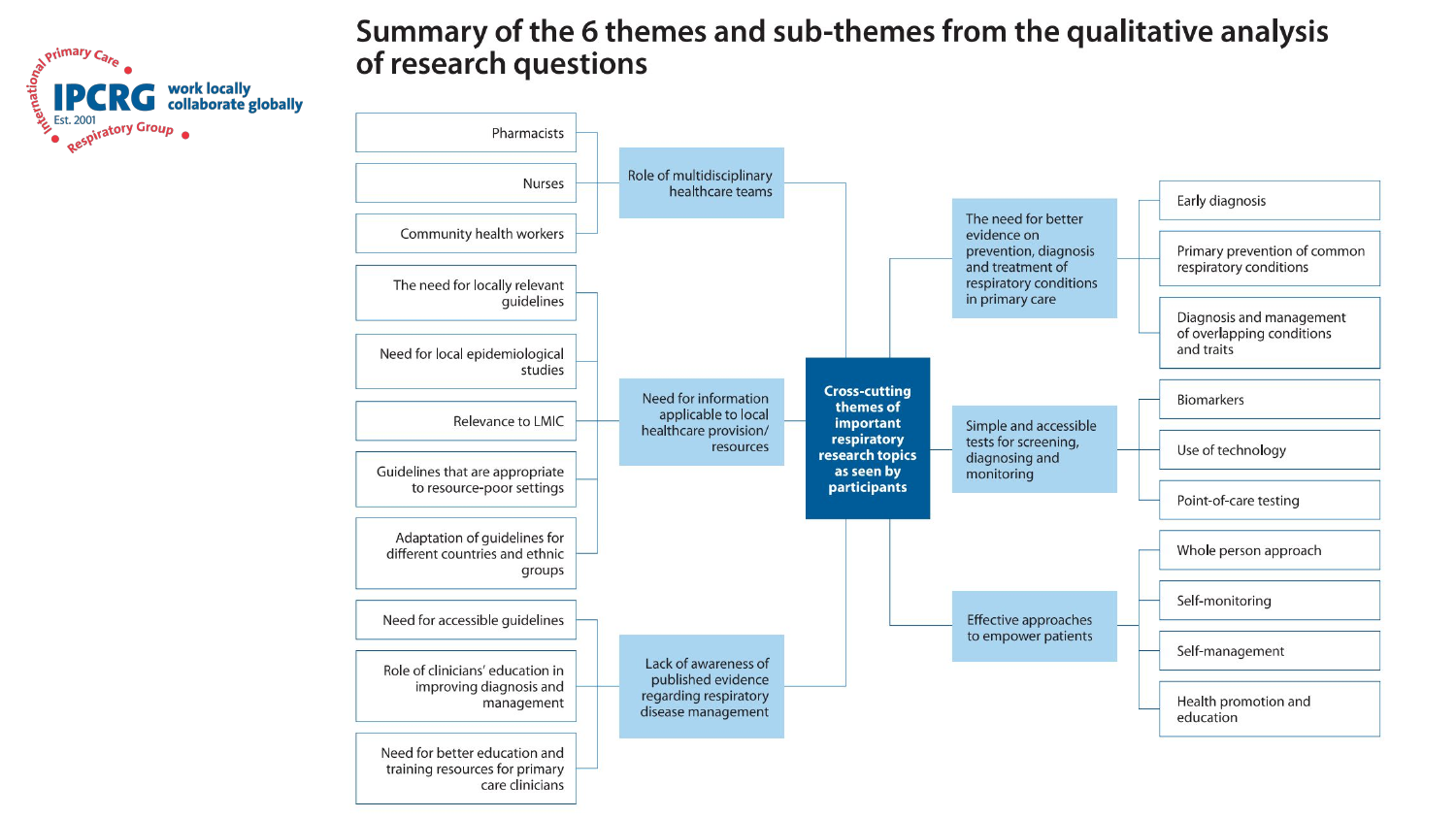

## Slide 13
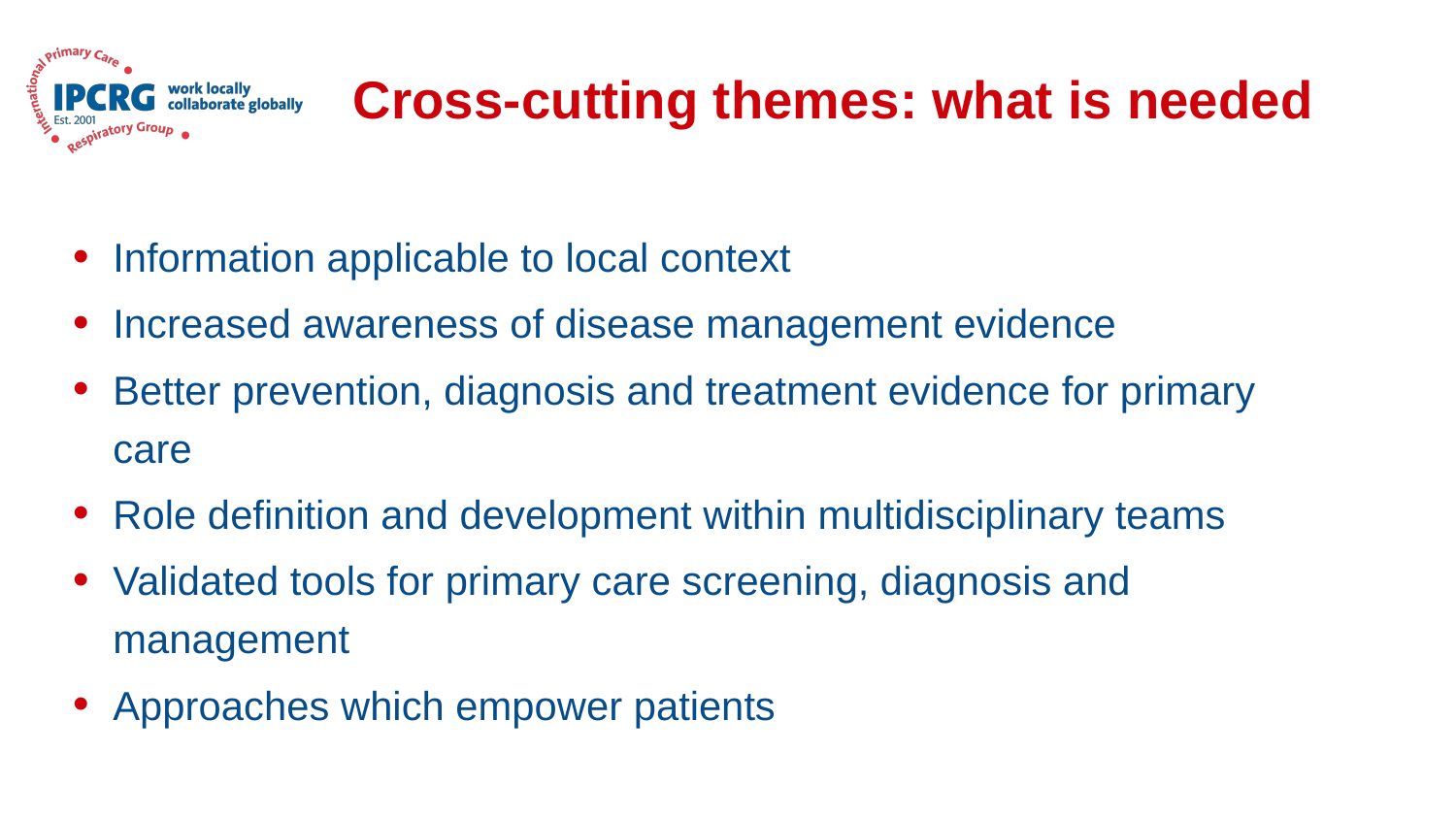

# Cross-cutting themes: what is needed
Information applicable to local context
Increased awareness of disease management evidence
Better prevention, diagnosis and treatment evidence for primary care
Role definition and development within multidisciplinary teams
Validated tools for primary care screening, diagnosis and management
Approaches which empower patients

## Slide 14
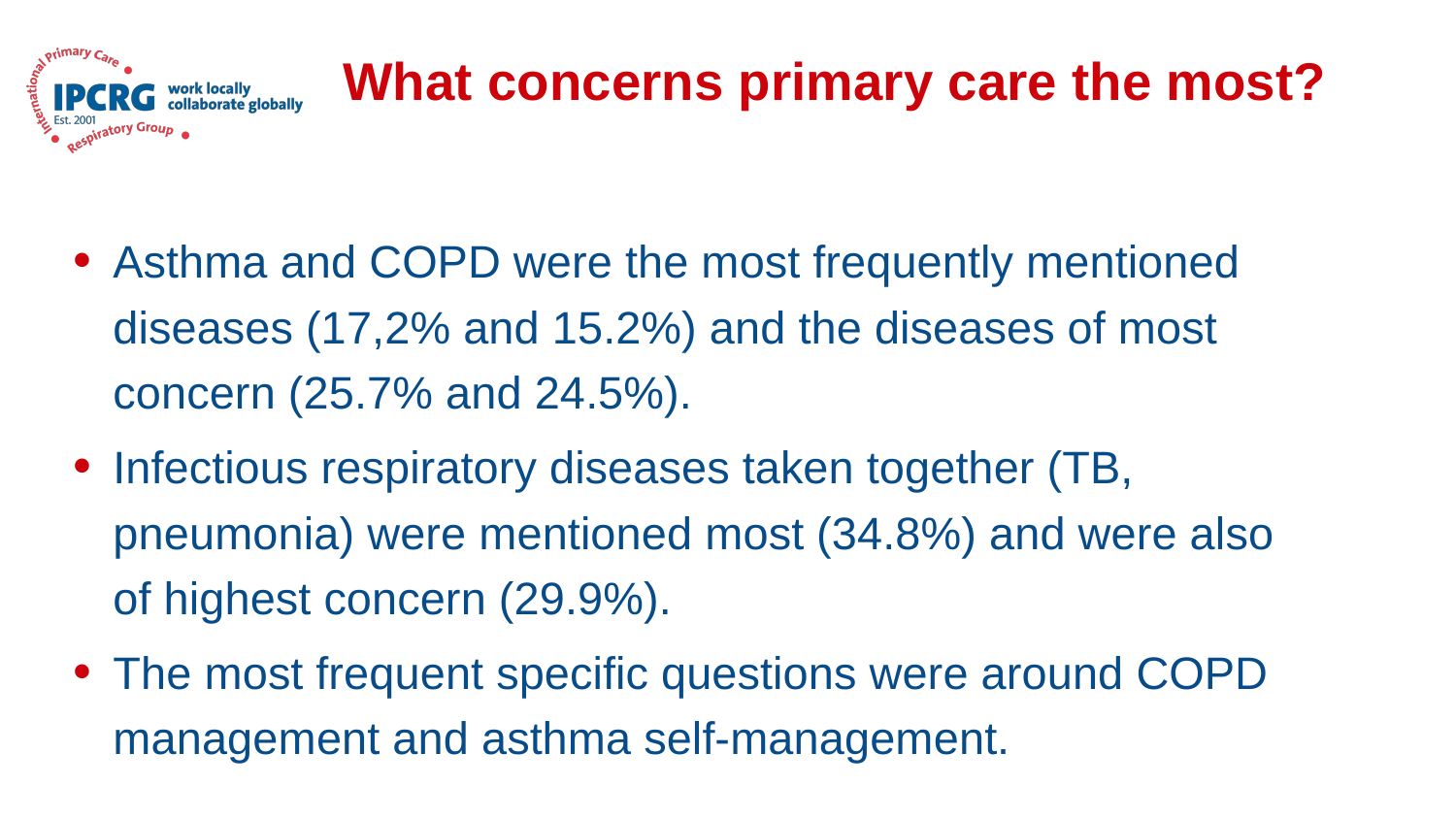

# What concerns primary care the most?
Asthma and COPD were the most frequently mentioned diseases (17,2% and 15.2%) and the diseases of most concern (25.7% and 24.5%).
Infectious respiratory diseases taken together (TB, pneumonia) were mentioned most (34.8%) and were also of highest concern (29.9%).
The most frequent specific questions were around COPD management and asthma self-management.

## Slide 15
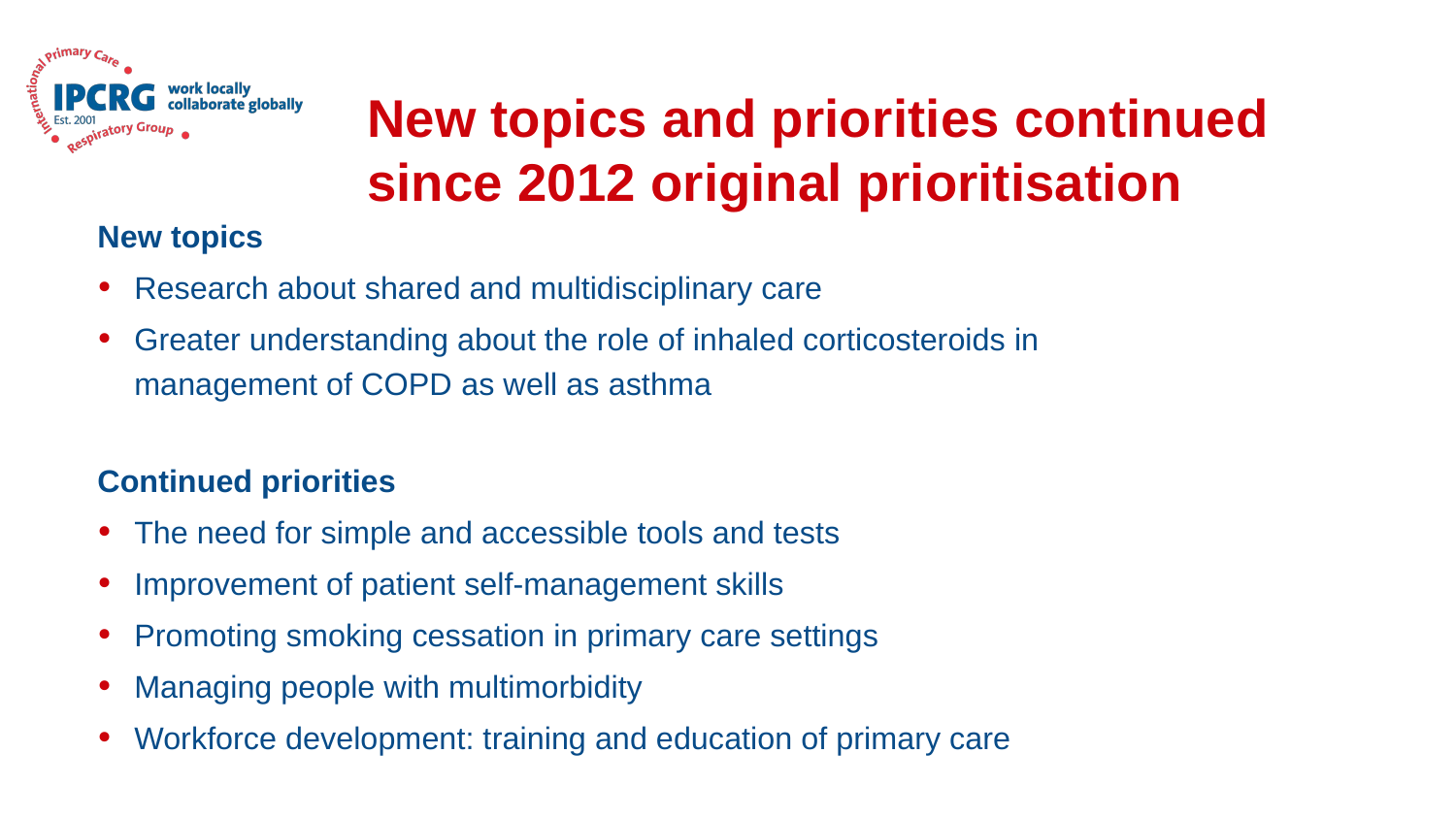

# New topics and priorities continued since 2012 original prioritisation
New topics
Research about shared and multidisciplinary care
Greater understanding about the role of inhaled corticosteroids in management of COPD as well as asthma
Continued priorities
The need for simple and accessible tools and tests
Improvement of patient self-management skills
Promoting smoking cessation in primary care settings
Managing people with multimorbidity
Workforce development: training and education of primary care

## Slide 16
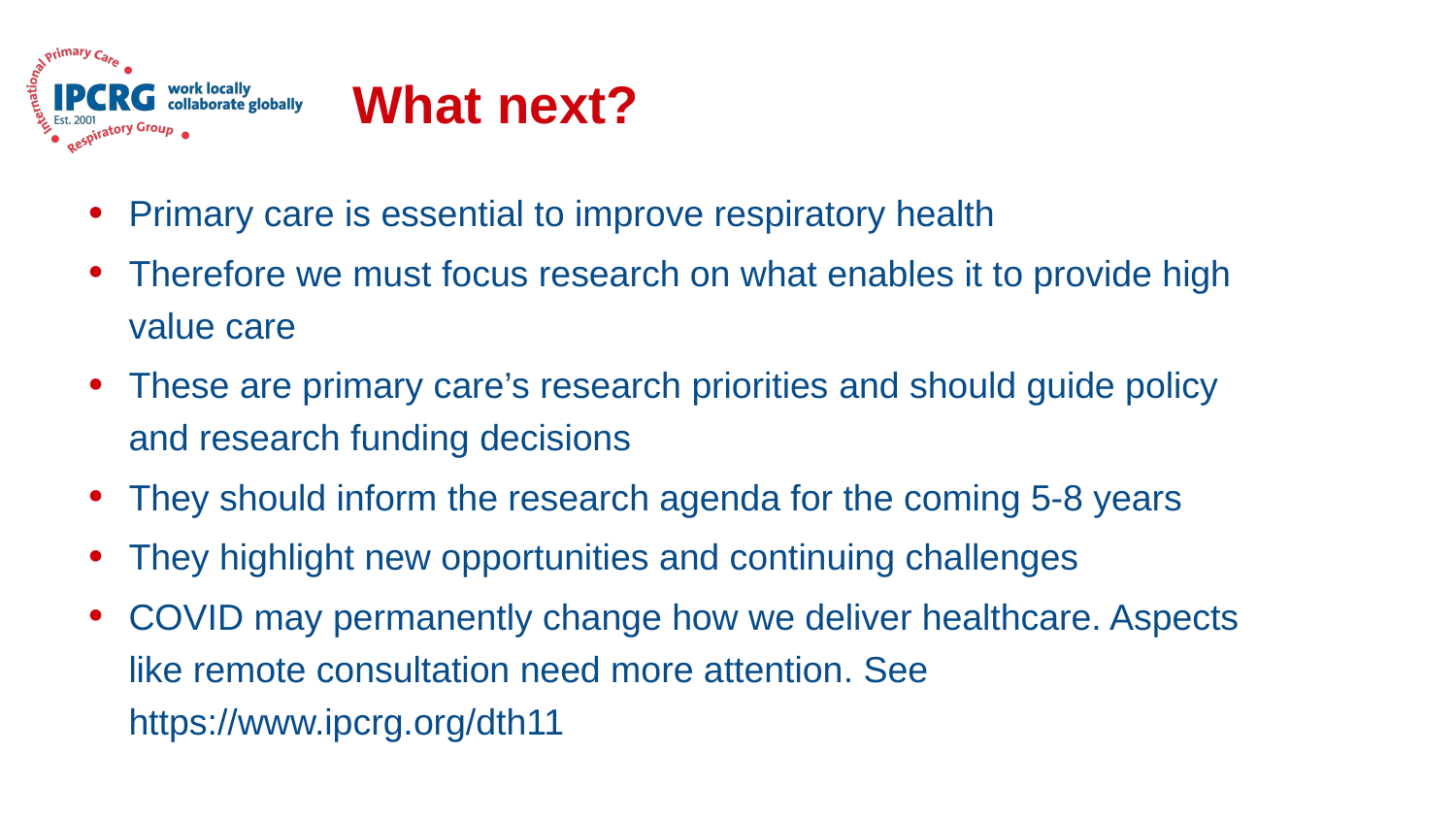

# What next?
Primary care is essential to improve respiratory health
Therefore we must focus research on what enables it to provide high value care
These are primary care’s research priorities and should guide policy and research funding decisions
They should inform the research agenda for the coming 5-8 years
They highlight new opportunities and continuing challenges
COVID may permanently change how we deliver healthcare. Aspects like remote consultation need more attention. See https://www.ipcrg.org/dth11
